# Hierarchical structures emerge from the cultural transmission: an iterated learning experiment using a non-linguistic task

**DOI:** 10.3389/frai.2023.1221329

**Published:** 2023-12-22

**Authors:** Seiya Nakata, Masanori Takezawa

**Affiliations:** ^1^Department of Behavioral Science, Graduate School of Humanities and Human Sciences, Hokkaido University, Sapporo, Japan; ^2^Japan Society for the Promotion of Science, Tokyo, Japan; ^3^International Research Center for Neurointelligence (WPI-IRCN), The University of Tokyo, Tokyo, Japan; ^4^Center for Experimental Research in Social Sciences, Hokkaido University, Sapporo, Japan; ^5^Center for Human Nature, Artificial Intelligence and Neuroscience, Hokkaido University, Sapporo, Japan

**Keywords:** cultural transmission, cumulative cultural evolution, emergent property, hierarchical structure, iterated learning, language evolution

## Abstract

Human language is characterized by complex structural features, such as the hierarchical combination of words to form sentences. Although other animals use communication systems, empirical evidence of hierarchical structures is rare. Computational studies of language evolution have suggested that cultural transmission plays a key role in the emergence of structural features in human languages, including hierarchy. While the previous study demonstrated the emergence of hierarchical structures in non-linguistic systems, we argue that their laboratory study may have overestimated the role of cultural transmission because of a lack of appropriate controls and analyses. To directly test the effect of cultural transmission, we conducted an experiment with no cultural transmission as a control (individual condition) in addition to replicating the previous transmission experiment (transmission condition). Our study has added a quantitative analysis of the hierarchical depth. We found that sequences became more structured as the number of generations increased; however, those produced under the transmission condition were more structured than those under the individual condition. These findings suggest that cultural transmission plays an important role in the emergence of hierarchical structures, which cannot be explained by increased learnability alone. The emergence of complex structural properties in human culture, such as language, technology, and music, may have resulted from information transmission processes between different individuals. In conclusion, this study provides evidence of the crucial role of cultural transmission in the emergence of hierarchical structures in non-linguistic communication systems. Our results contribute to the ongoing debate on the origins of human language and the emergence of complex cultural artifacts. The results of this study have implications for the study of cultural evolution and the role of transmission in shaping the emergence of structural features across diverse domains.

## 1 Introduction

The most remarkable feature of human language is its systematic structure. For example, human languages generate words, phrases, and sentences by merging morphemes, words, and phrases, respectively. Through the recursive merging of sub-elements, human languages express meanings that cannot be transmitted merely by listing the sub-elements (Tamariz and Kirby, 2015; Fujita, 2017). Animals other than humans have rarely been reported to use hierarchical signals to communicate with others (Hauser et al., 2002; Fitch and Hauser, 2004; but see also Suzuki et al., 2020). In humans, not only language, but also other cultures, such as music and stone toolmaking, have hierarchical structures similar to language (Stout, 2011; Asano and Boeckx, 2015; Asano, 2022) and the ability to generate hierarchical structures may contribute to the production of various solutions (Toya and Hashimoto, 2018).

Computational evolutionary linguists argue that language structure is an emergent property of the iterative process of learning from and transmitting to others (Kirby et al., 2007). Researchers have used an iterated learning approach to demonstrate the cumulative cultural evolution of language structures (Kirby et al., 2008). Iterated learning is designed to replicate the process of language transmission from parents to children. When children learn language from adult utterances, they are not explicitly taught systematic grammatical rules but are given a limited set of utterances. Children infer grammatical rules from a limited set of adult utterances and speak based on these inferences (Hsu and Griffiths, 2016). As children grow up, the next generation receives and infers language from the previous generation. Many studies applying iterated learning to computational models and laboratory experiments have shown that random sequences of characters (artificial languages) gradually become learnable through learning and transmission processes between generations, resulting in a systematically structured language (Brighton et al., 2005; Kirby et al., 2007). Thus human language may have evolved culturally such that children can learn language from limited examples.

However, the language structures that emerge in the laboratory are heavily influenced by the experimental framework and the participants' prior language biases (Kirby, 2017). The participants were informed that the goal of learning was language. They were shown many alphabetic sequences and were asked to recall them after learning. Of course, they learn natural language through a systematic structure. Thus, they were able to apply their natural language structure to the experimental task, and the structures that emerged in the laboratory could be artifacts of the participants' intentional design. However, the emergence of a systematic structure can be explained as a general property of iterated learning, and not just a language bias. Human cultures other than language have hierarchical structures similar to those of language, as discussed above (Stout, 2011; Asano and Boeckx, 2015; Asano, 2022). Cornish et al. designed an iterated learning experiment using a non-linguistic task (Cornish et al., 2013). They developed a task based on *the Simon Game*, an electronic game for children. The participants were asked to remember the order of the flashed color sequence on a tablet device and to reproduce the sequence immediately after it was displayed. One participant remembered and reproduced the 60 sequences, which were transmitted to a next-generation participant following the iterated learning paradigm. As a result of ten generations of iterated learning, randomly ordered sequences (seed sequences) gradually became learnable and structured. Although their study lacked the appropriate control condition and a quantitative analysis of the hierarchical structure, it had several implications. Their findings suggest that the cultural selection of learnability generally leads to the emergence of a systematic structure. In other words, the emergence of systematic structures such as hierarchies in human culture could be explained by a single common process.

Given the important implications of Cornish et al. (2013) regarding the cumulative cultural evolution of a systematic structure, improving their experimental design and analysis is valuable. To test the validity of the argument that systematic structures emerge through iterated learning, we modified the work of Cornish et al. (2013). Two major changes were introduced. First, we added a control condition with no cultural transmission (individual condition). To assess the contribution of cultural transmission beyond individual cognitive processes, we compared the individual condition with the transmission condition by replicating the work of Cornish et al. (2013). The procedure for cultural transmission was identical to Cornish's experiments. Second, we added a quantitative analysis of the emergence of the hierarchical structure. Previous studies qualitatively analyzed the structures that emerged in their experiments and found hierarchical structures. We applied grammar compression algorithms to extract the depth of the hierarchy from the sequences. If the cumulative cultural evolution of a structure is a general phenomenon, not only language but also cultural transmission contributes to the emergence of structures in sequences.

## 2 Methods

We confirmed the robustness of our findings by conducting two experiments using slightly different procedures. Unless otherwise stated, the methods used in the two experiments were identical.

### 2.1 Participants

#### 2.1.1 Experiment 1

A total of 120 undergraduate and graduate students from Hokkaido University (36 female, 84 male; mean age 20.00 years, age range 18–23) were recruited. One hundred participants were assigned to the transmission condition and 20 to the individual condition; each participant experienced only one condition.

#### 2.1.2 Experiment 2

A total of 180 undergraduate and graduate students at Hokkaido University (74 female, 106 male; mean age 20.55 years, age range 18–23) were recruited. A total of 150 participants were assigned to the transmission condition and 30 to the individual condition; the participants experienced only one of the conditions.

### 2.2 Task

**Figure 1:**
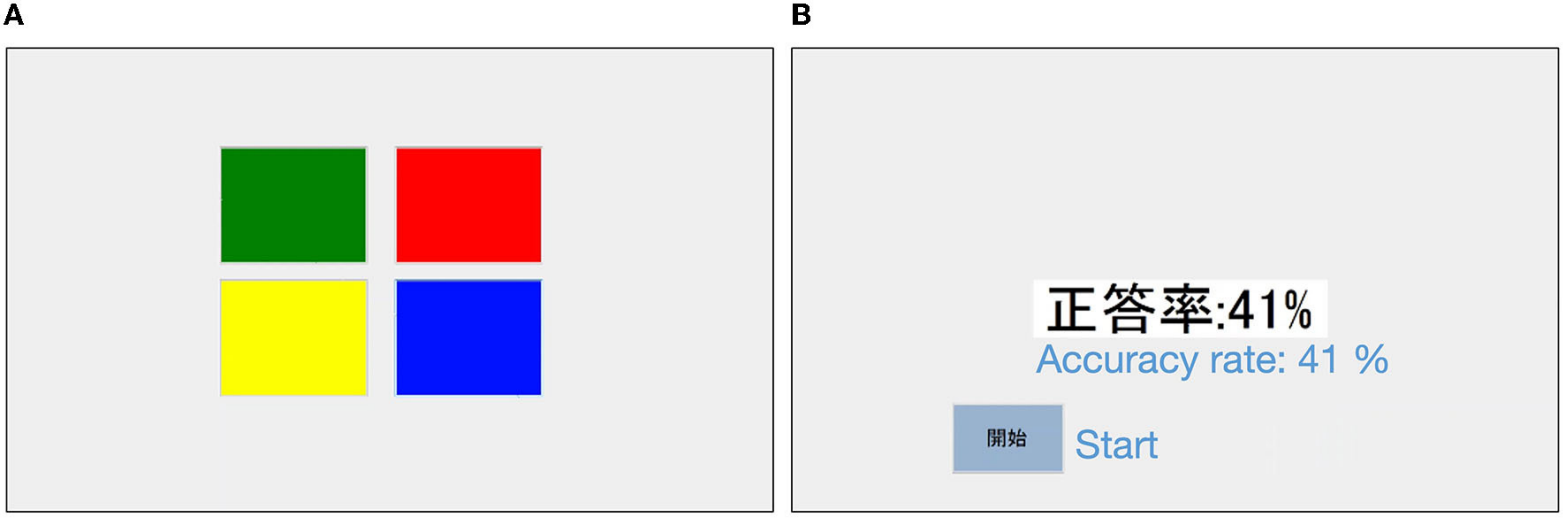
Sample screenshot of the experiment. **(A)** Is the screen where the participant tapped to reproduce a sequence. **(B)** Is a screen with feedback on the accuracy of reproduction. English translations of the labels are added.

In this experiment, participants performed the Simon Game task (Cornish et al., 2013), which required them to memorize a sequence of stimuli that combined four colors (Figure 1). Participants tapped the “start” button on a tablet and were presented with a sequence of four colors (red, green, yellow, and blue) that flashed in sequence. The length of all the presented stimulus sequences was 12. After the presentation, the participants were asked to reproduce the sequence in the order presented by tapping the corresponding color fields on the tablet. Once tapped, participants could not go back, and after a total of 12 taps, a “Done” button appeared. After tapping the “Done” button, the correct rate of the sequence reproduced by the participant (details for calculation described below) was presented. The process from presenting the sequence to displaying feedback at the correct rate is one trial. Participants could proceed to the next trial by tapping the “Start” button again. The experimental task was created using Visual Basic 2017 (version 15.0) software.

### 2.3 Procedure

**Figure 2:**
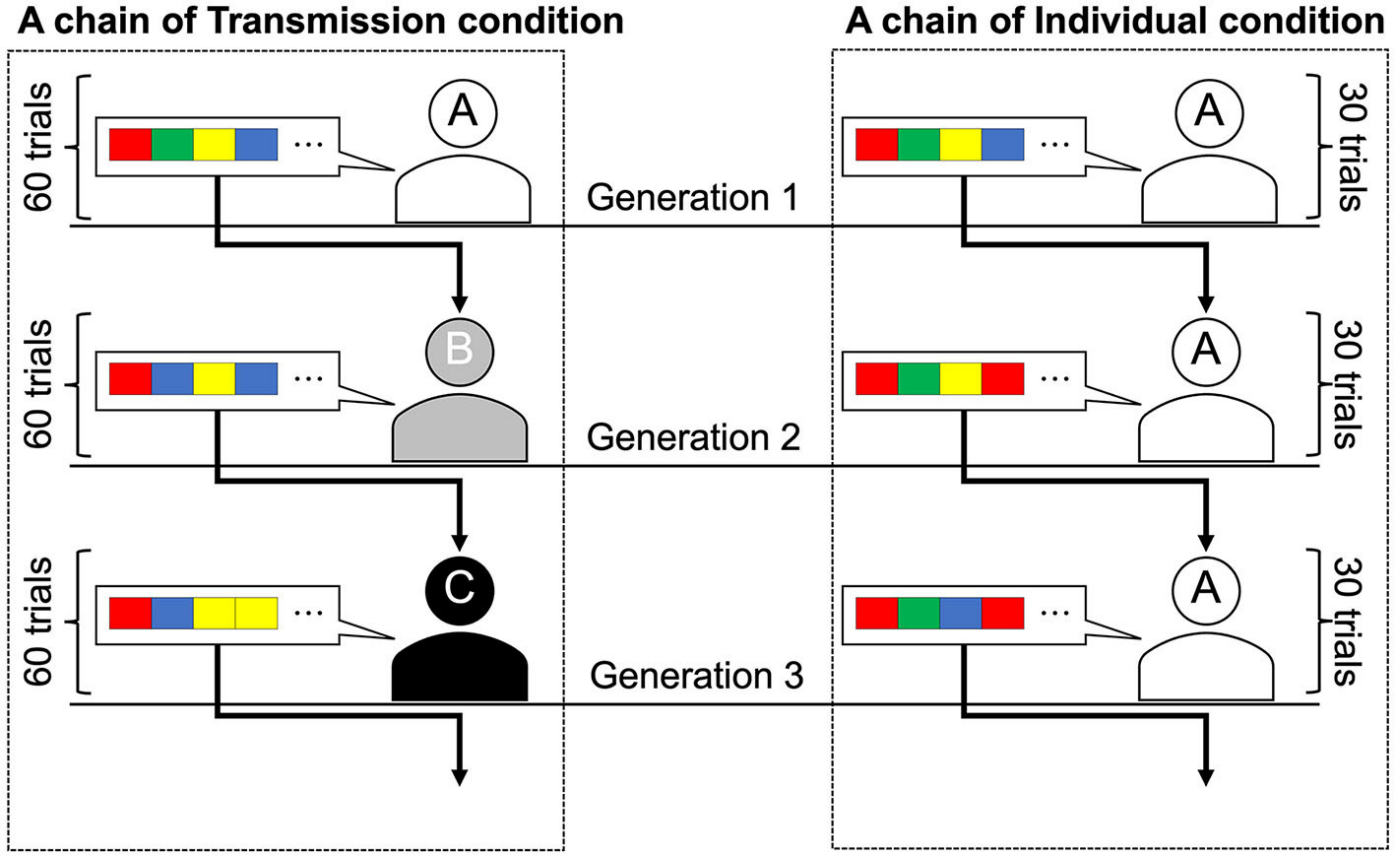
Graphical representations of the two conditions. Transmission condition: Generation 1 participant learned the sequence order and were asked to reproduce it. The number of trials of the task was 60 per generation, and different sequences were presented between trials. Sequences reproduced by the Generation 1 participant were transmitted to the Generation 2 participant as a learning set. This learning and transmission procedure continued until Generation 10. Individual condition: The same participants were assigned to all generations, so there was no actual generational change. The number of trials of the task was 30 per generation. The sequences reproduced by the participant in the first 30 trials (Generation 1) were presented to the same participant in the next 30 trials (Generation 2). This procedure continued until Generation 10 (i.e., participants worked on 300 trials).

Participants worked on the tasks individually while seated in separate booths. In the iterated learning experiments, after the first participant completes the task, the second participant receives the output of the first participant and works on the task. In experiments that replicate cultural transmission, the first participant is called the first generation of participants, and a sequence of participants is called a (transmission) chain. Each transmission chain can be considered a different society or social group in which a unique culture evolves independently. The transmission condition was a replication of Cornish et al. (2013), who used an iterated learning design to simulate intergenerational cultural transmission in the laboratory (Figure 2). Under this condition, each participant completed a 60-trial task, with the first generation receiving a randomly generated stimulus sequence. Subsequent generations were presented with 60 stimulus sequences generated by the previous generation of participants who completed the task. The participants were not informed that the stimulus sequences were generated by other participants. Ten chains of ten transmission generations were used in this experiment.

The participants in the individual condition completed a total of 300 trials, that is, 30 trials per generation for 10 generations. In the first 30 trials of this condition, the participants were presented with 30 randomly selected stimulus sequences from the 60 sequences presented to the first generation in the transmission condition and reproduced them. For trials 31–60, the participants were presented with the sequences they had reproduced in trials 1–30. Similarly, every 30 trials, participants were presented with the stimulus sequences they generated in the previous 30 trials so that each participant in the individual condition completed 10 generations of the task. Participants in the individual condition were not informed that they would be presented with their own stimulus sequences. To prevent participants from noticing this manipulation, they were instructed to take breaks of at least 10 seconds after every 50 trials. The post-trial questionnaire responses showed that none of the participants were aware that they were being presented with their own stimulus sequences. To ensure that the number of stimulus sequences per generation was equal across all chains and conditions, we set the number of sequences per generation to 30 in the individual condition and ran the experiment with 20 chains, which was twice the number used in the transmission condition. In both conditions, the order in which the stimulus sequences were presented was counterbalanced across the transmission chains and generations.

The reward amounts were based on the participants' performance and calculated from the average percentage of correct responses across all tasks. Because participants in the individual condition needed more time to complete the task, we decided to use two different pay scales for the two conditions in order to keep the hourly pay comparable. In the transmission condition, participants were paid 120 JPY (approximately 0.8 EUR/0.9 USD) per 10% average correct response rate, with a minimum compensation of 120 JPY for an average correct response rate of 0–10%, and a maximum of 1,200 JPY for an average correct response rate of 91–100%. Similarly, the individual condition paid 450 JPY per 10% average correct response rate, with a minimum of 450 JPY for an average correct response rate of 0–10%, and a maximum of 4,500 JPY for an average correct response rate of 91–100%.

In Experiment 1, the order of presentation of the stimulus sequences was fixed across chains and generations. In the individual condition, the stimulus sequence that the participant reproduced in the first trial of the first generation was presented in the first trial of the second generation (Trial 31), and the stimulus sequence that the participant reproduced in the second trial of the first generation was presented in the second trial of the second generation (Trial 32). In Experiment 2, the order in which the stimulus sequences were presented was randomized and modified to differ between generations and transmission chains.

### 2.4 Analysis

For quantitative analysis, four-color sequences were converted into strings. Each color, red, green, blue, and yellow, was converted to “r,” “g,” “b,” and “y” string data, respectively. We measured the same dependent variables as in Cornish et al. (2013): the accuracy of reproduction, compression ratio for structure, and diversity of sequences across transmission chains. While Cornish et al. (2013) performed a qualitative analysis of hierarchical structures, we applied a grammatical compression algorithm to quantitatively analyze the depth of the hierarchy. To examine the effect of cultural transmission on each measure, two multilevel Bayesian (beta regression) models were constructed. In Model 1, the dependent variable was each measure (accuracy, compression ratio, depth of hierarchy, or diversity), the independent variables were condition (0: transmission condition, 1: individual condition) and generation, and the random intercepts were the transmission chain number and stimulus sequence number. In Model 2, the interaction between the condition and generation was added to Model 1 as an independent variable. All models were analyzed using the rethinking package (McElreath, 2020) ver. 2.31 (https://github.com/rmcelreath/rethinking). Results were evaluated by analyzing the effects of the variables, and checking the adequacy of the analytical model; each model was evaluated in terms of the estimated parameter values and 89% credible intervals, model comparison using WAIC, and posterior predictive distribution. We checked whether the 89% credible intervals of the model parameters did not cross zero. A total of 89% credible intervals are the default in the Rethinking package and are used to prevent researchers from inadvertently confusing the results with null hypothesis significance tests (McElreath, 2020). For model comparisons, we compared the WAIC value of the models. Lower WAIC value indicates that it was considered a better model. Following McElreath (2020), we evaluate the effect of variables by combining these two criteria. Posterior predictive distributions include uncertainty in the estimated parameter, uncertainty in the prediction, and checking the posterior predictive distributions is useful for verifying the adequacy of the statistical model. See the Supplementary material for details on the model formulae used in each analysis.

#### 2.4.1 Accuracy

Cornish et al. (2013) reported that the participants' accuracy gradually increased across generations in their transmission chains. To measure the accuracy, we calculated the normalized edit distance between sequences (Levenshtein, 1966). The edit distance between two sequences is the minimum number of changes, that is, insertions, deletions, or substitutions. For example, the string ABC can be converted into an ADCD by replacing B with D and inserting D at the end of the string. In other words, the editing distance is 2.

In this study, we calculated the standardized edit distance, which is the edit distance divided by the maximum length of the string, and the value 1- standardized edit distance was used as the accuracy measure. If the sequence reproduced by the participant perfectly aligned with the presented sequence, the accuracy took the maximum value of 1. During the experimental task, accuracy was expressed as a percentage (rounded down to the nearest whole number) and fed back to the participants.

#### 2.4.2 Compression ratio

Cornish et al. (2013) measured the compression ratio of a sequence as an indicator of the structure. We used GZIP to compress the text file stored in each sequence and calculated the compression ratio (file size after compression/file size before compression). If the distribution of strings is skewed, such as repetition of some chunks (e.g., “rgb-rgb-rgb-rgb”), the text file is compressed smaller. In other words, a smaller value indicates that the sequence has a non-random structure. Note that compression ratio is an indirect indicator of the structure and cannot identify the type of structure.

#### 2.4.3 Depth of hierarchy

Cornish et al. (2013) reported that hierarchical structures emerged in sequences consisting of several chunks, such as “[ry]-[ry]-[rbyg]-[rbyg],” as a result of 10 generations of cultural transmission. This suggests the possibility of the cumulative cultural evolution of hierarchical structures. In this study, we conducted a new analysis using a grammar-compression algorithm to quantitatively evaluate the cumulative cultural evolution of hierarchical structures. To extract the hierarchical structure within sequences, we used “sequitur,” a grammar compression algorithm (Nevill-Manning and Witten, 1997). “Sequitur” repeats the process of assigning a new string to the pattern of two adjacent characters in the target string, replacing it with a shorter string with a hierarchical structure. In this study, we measured the depth of the hierarchical structure extracted by “sequitur.”

#### 2.4.4 Diversity

Cornish et al. (2013) found that the transmission of stimulus sequences increased the diversity among the transmission chains as generations progressed, and that different structures emerged for each chain. To measure diversity, we calculated the within-chain and across-chain similarities of sequences using edit distance and accuracy. Within-chain similarity is the average similarity of reproduced sequences from a participant in a chain. Across-chain similarity is the average similarity between reproduced sequences from one participant in a chain and reproduced sequences from other chains of participants of the same generation. Diversity was calculated as [within-chain similarity]/[within-chain similarity + across-chain similarity] and ranged from 0 to 1. A diversity value >0.5 indicates that the within-chain similarity is relatively higher than the across-chain similarity, i.e., the diversity between chains is higher.

## 3 Results of Experiment 1

### 3.1 Accuracy

**Figure 3:**
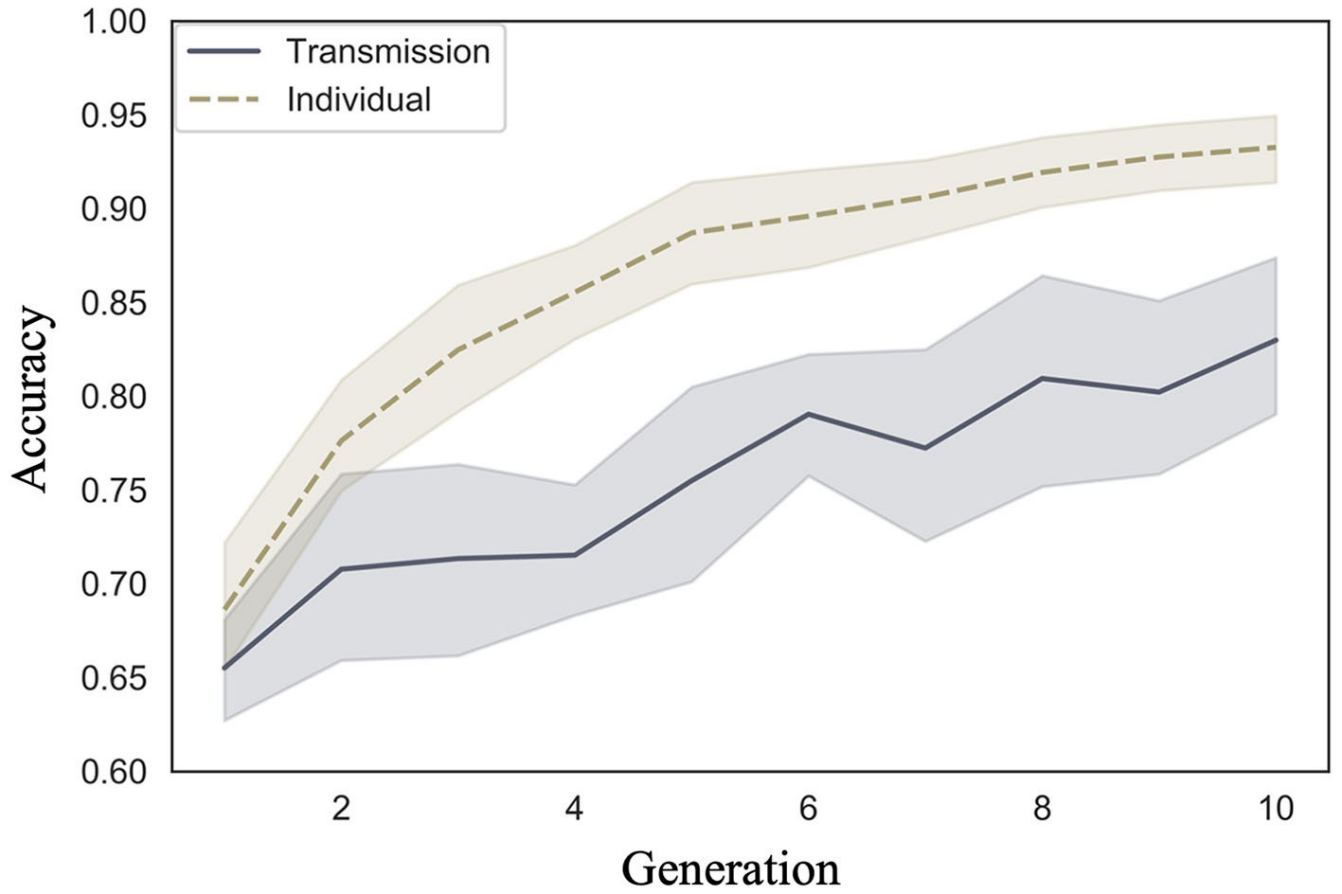
Changes in mean accuracy (10 chains in transmission chain; 20 chains in individual condition) over the course of ten generations in Experiment 1.

We examined whether accuracy increased across generations in the transmission condition, a replication of Cornish et al. (2013); whether accuracy also increased in our individual condition; and whether there were differences in the rate of increase across conditions. Figure 3 shows the mean accuracy values and 95% confidence intervals (calculated on 1,000 bootstrap samples using the Python seaborn package ver. 0.11.2) under each condition and generation. In both conditions, the accuracy increased as the number of generations increased.

Model 2, which included an interaction term, showed a smaller WAIC (−83,932.82) than Model 1, which did not include an interaction term (WAIC = −83,912.29), indicating a higher predictive power. The difference in the WAIC between the two models was 20.53 (dSE = 10.62), which is sufficiently large. The Bayesian estimation results for Model 2 (Table 1) show that the 89% confidence intervals for the condition, generation, and interaction terms did not include 0 (condition: β = 0.51, CI = [0.24, 0.77]; generation: β = 0.16, CI = [0.15, 0.17]; interaction: β = 0.04, CI = [0.02, 0.05]). That is, the accuracy improved with each successive generation in both conditions, but the improvement was greater in the individual condition.

**Table 1:** Bayesian estimated values for Model 2 (with interaction) with accuracy in Experiment 1 as dependent variable.

	**β**	***Std***	***Lower 0.89***	***Upper 0.89***	***n_eff***	***Rhat***
Intercept	0.67	0.14	0.45	0.88	3,244.10	1.00
Condition	0.51	0.17	0.24	0.77	2,785.34	1.00
Generation	0.16	0.01	0.15	0.17	28,368.87	1.00
Interaction	0.04	0.01	0.02	0.05	27,973.73	1.00

**Figure 4:**
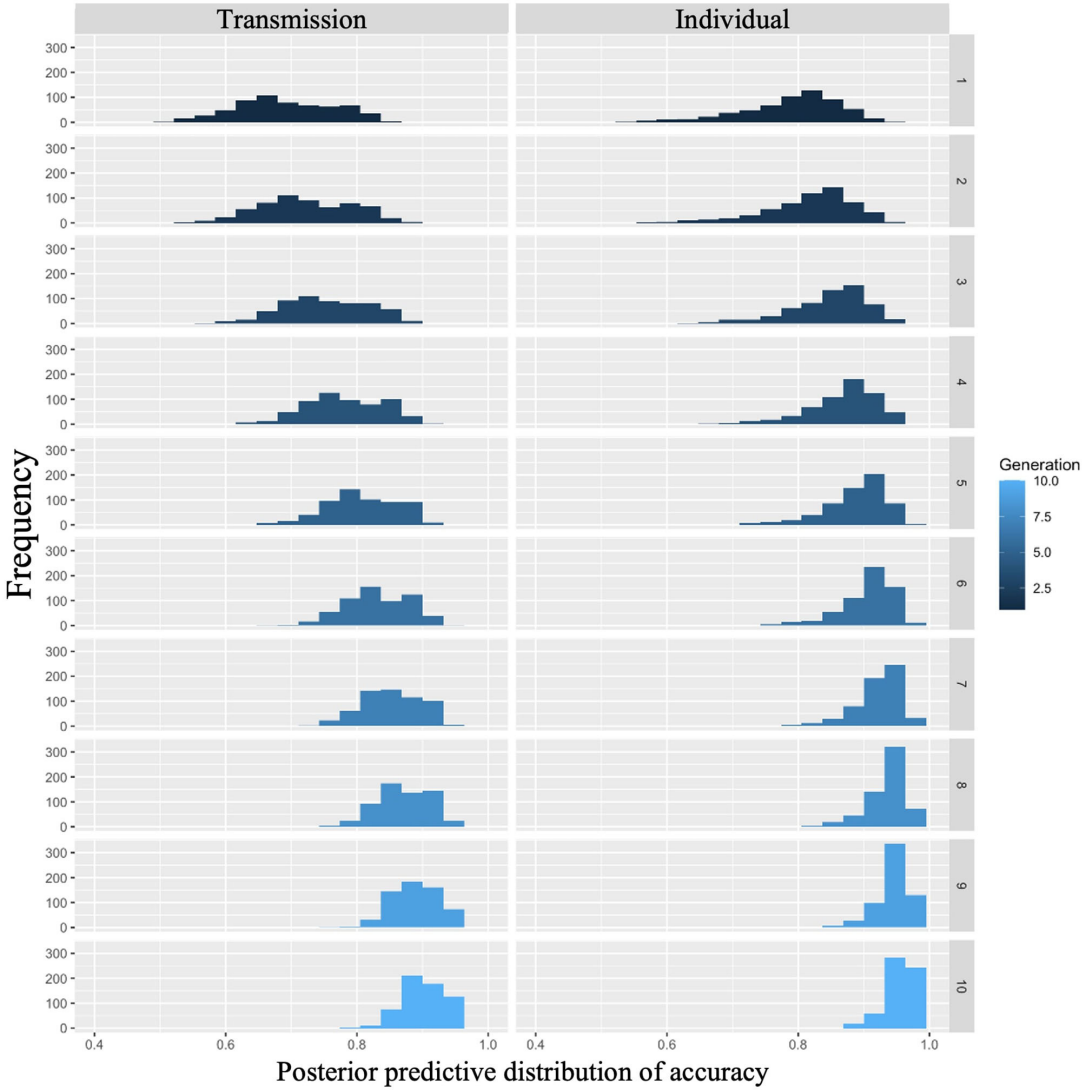
Accuracy predicted from posterior distribution in Experiment 1. The **left** column shows the transmission condition, and the **right** column shows the individual condition. Rows indicate generations.

The posterior predictive distribution for Model 2 with interactions is shown in Figure 4, where the rows indicate generations (1–10) and the columns indicate conditions (0: transmission condition, 1: individual condition). The horizontal and vertical axes of each plot indicate accuracy and frequency, respectively. The distribution of posterior predictions for the first generation shows that the transmission condition is slightly more accurate than the individual condition, but there is not much difference. As generations progressed, both conditions became more accurate, but the individual conditions showed higher values. For example, in the 10th generation, the mode of the histogram in the individual condition was 0.95, while it was < 0.9 in the transmission condition.

### 3.2 Compression ratio

**Figure 5:**
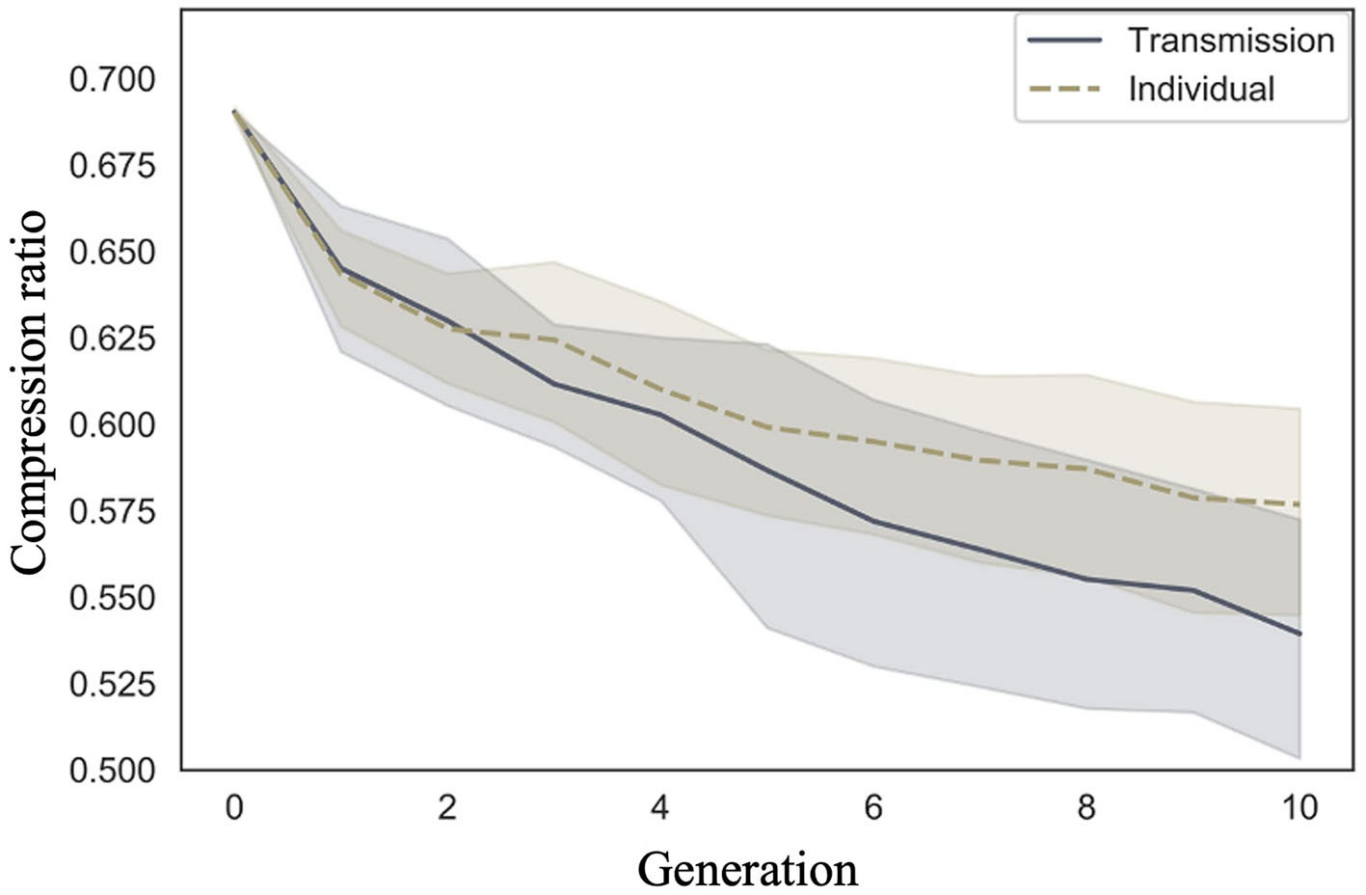
Changes in mean compression ratio (10 chains in transmission chain; 20 chains in individual condition) over the course of ten generations in Experiment 1.

We examined whether compression ratio decreased across generations in the transmission condition, a replication of Cornish et al. (2013); whether compression ratio also decreased in our individual condition; and whether there were differences in the rate of decrease across conditions. Figure 5 shows the mean compression ratio values and 95% confidence intervals under each condition and generation. Under both conditions, the compression ratio decreased as the number of generations increased.

Model 2, which included an interaction term, showed a smaller WAIC (−14,641.87) compared to Model 1, which did not include an interaction term (WAIC = −14,602.55), indicating a greater predictive power. The difference in WAIC between the two models was 39.32 (dSE = 16.58), which is sufficiently large. The Bayesian estimation results in Model 2 (Table 2) show that the 89% confidence intervals for the generation and interaction terms did not include 0 (generation: β = −0.06, CI = [−0.07, −0.06]; interaction: β = 0.02, CI = [0.02, 0.03]). In other words, the compression ratio decreased with increasing generations under both conditions; however, the degree of decrease was greater under the transmission condition.

**Table 2:** Bayesian estimated values for Model 2 (with interaction) with compression ratio in Experiment 1 as dependent variable.

	**β**	***Std***	***Lower 0.89***	***Upper 0.89***	***n_eff***	***Rhat***
Intercept	0.66	0.08	0.52	0.79	5,251.99	1.00
Condition	−0.04	0.10	−0.20	0.12	4,707.90	1.00
Generation	−0.06	0.00	−0.07	−0.06	56,584.82	1.00
Interaction	0.02	0.00	0.02	0.03	60,522.84	1.00

**Figure 6:**
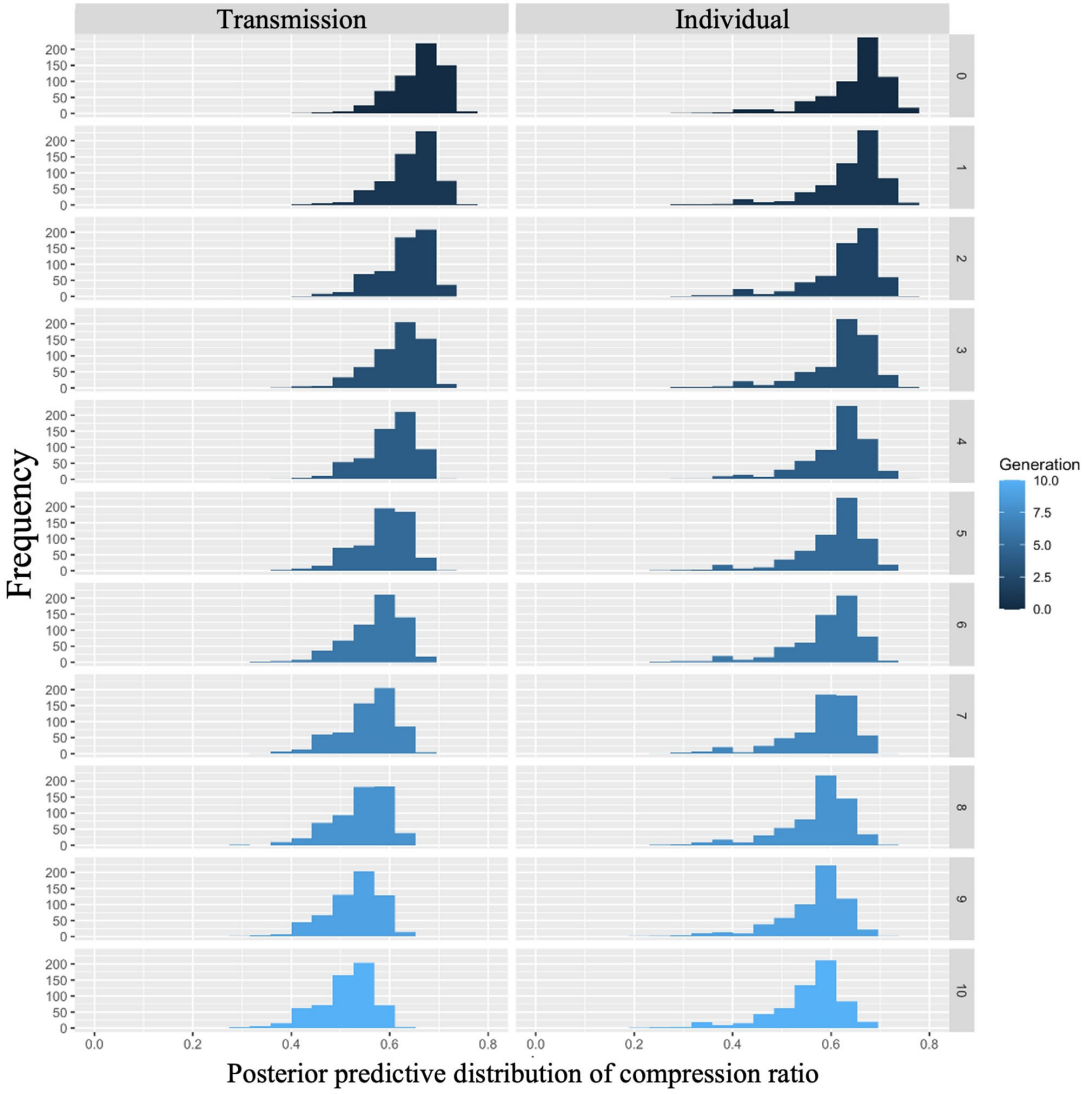
Compression ratio predicted from posterior distribution of Model 2 in Experiment 1. The **left** column shows the transmission condition, and the **right** column shows the individual condition. Rows indicate generations.

The posterior predictive distribution of the model with interactions is shown in Figure 6. The rows in the figure indicate generations (1–10) and the columns indicate conditions (0: transmission condition, 1: individual condition). The vertical axis of each graph shows the frequency, and the horizontal axis represents the compression ratio. There was no clear difference in the posterior predictive distribution of the compression ratios in the first generation. As the generation progressed, the compression ratio decreased in both conditions, but less so in the transmission condition. For example, in the 10th generation, the mode of the histogram for the individual condition was 0.65, while it was 0.55 in the transmission condition.

### 3.3 Depth of hierarchy

**Figure 7:**
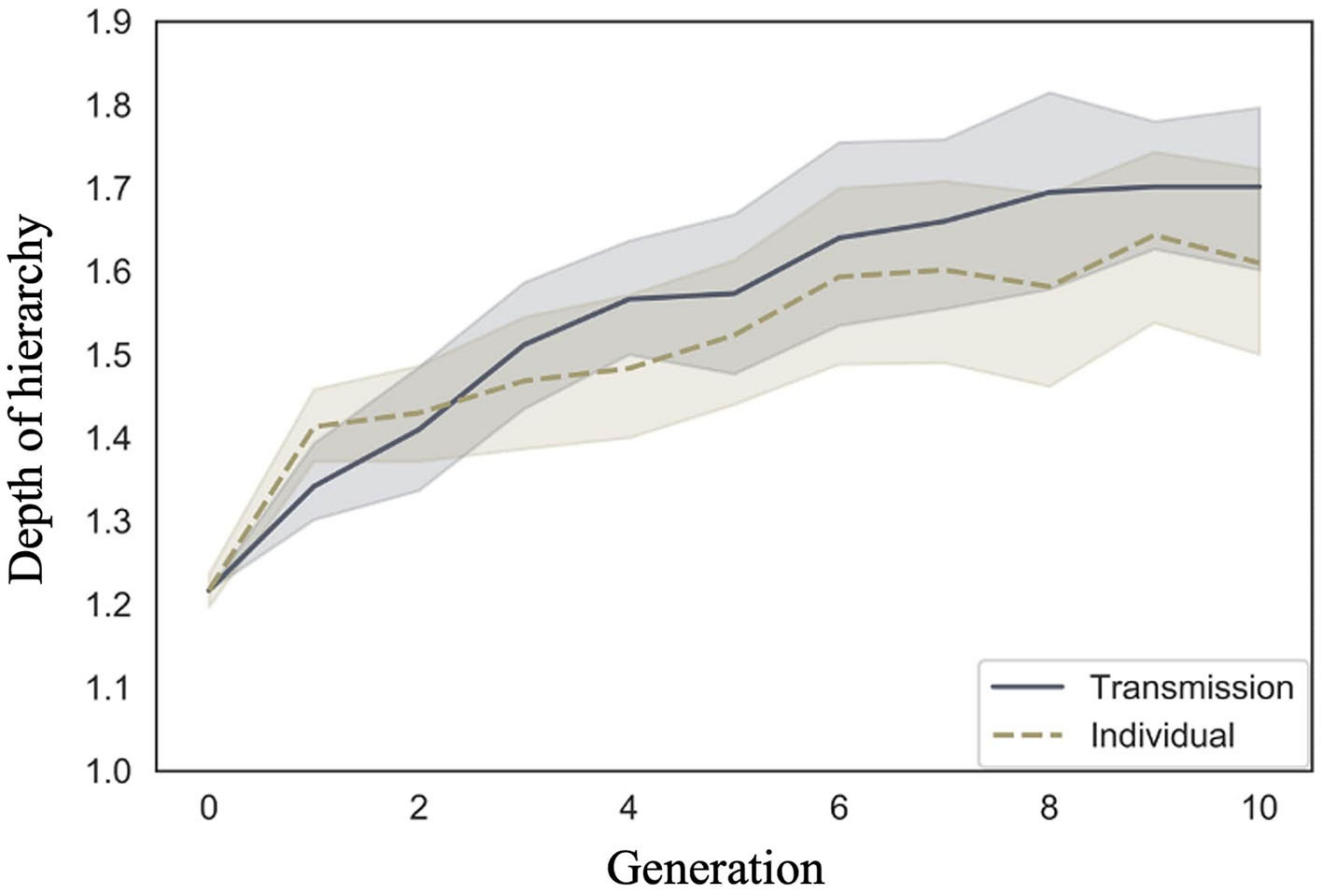
Changes in mean depth of hierarchy (10 chains in transmission chain; 20 chains in individual condition) over the course of ten generations in Experiment 1.

Cornish et al. (2013) qualitatively analyzed the emergence of hierarchical structures, whereas we quantitatively analyzed their depth. We examined whether the depth of the hierarchy increased across generations in both conditions, and whether there were differences in the rate of increase across conditions. Figure 7 shows the mean depth of the hierarchy and 95% confidence intervals under each condition and generation. Under both conditions, the depth of the hierarchy increases with the number of generations.

The WAIC (34,171.12) for Model 2 with the interaction included was smaller than the WAIC (34,172.46) for Model 1 without the interaction, indicating greater predictive power. The difference in WAIC between the two models was 1.33 (dSE = 1.83), a small difference. The Bayesian estimation results for Model 2 (Table 3) showed that the 89% confidence intervals for the generation and interaction terms did not include zero (generation: β = 0.03, CI = [0.02, 0.04]; interaction: β = −0.01, CI = [−0.01, −0.00]). In other words, the hierarchy becomes deeper as generations progress in both conditions but more so in the transmission condition.

**Table 3:** Bayesian estimated values for Model 2 (with interaction) with the depth of hierarchy in Experiment 1 as dependent variable.

	**β**	***Std***	***Lower 0.89***	***Upper 0.89***	***n_eff***	***Rhat***
Intercept	0.28	0.04	0.22	0.34	6,517.67	1.00
Condition	0.01	0.05	−0.06	0.08	6,782.07	1.00
Generation	0.03	0.00	0.02	0.04	34,134.93	1.00
Interaction	−0.01	0.00	−0.01	−0.00	32,119.27	1.00

**Figure 8:**
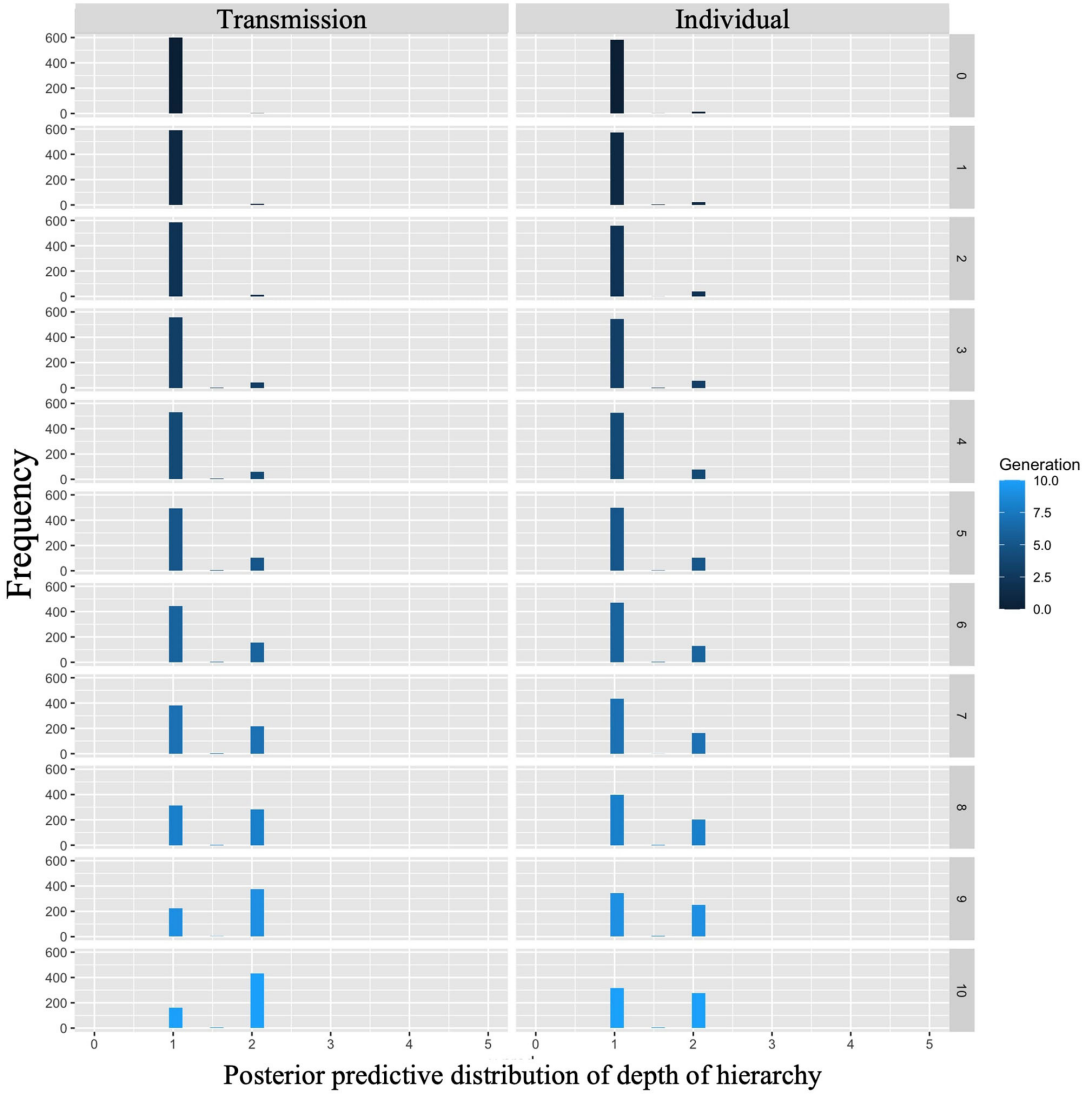
Depth of hierarchy predicted from posterior distribution of Model 2 in Experiment 1. The **left** column shows the transmission condition, and the **right** column shows the individual condition. Rows indicate generations.

The posterior predictive distribution for the model with interactions is shown in Figure 8. The rows in the figure indicate generations (1–10) and the columns indicate conditions (0: transmission condition, 1: individual condition). The vertical axis of each plot indicates the frequency, and the horizontal axis indicates the depth of the hierarchy. The posterior predictive distribution of the hierarchy depth in the first generation showed little difference. As the generations progress, the hierarchy becomes deeper under both conditions, but the transmission condition shows a greater increase. For example, in the 10th generation, the mode of the histogram for the individual condition was 1, while it was 2 in the transmission condition.

### 3.4 Diversity

**Figure 9:**
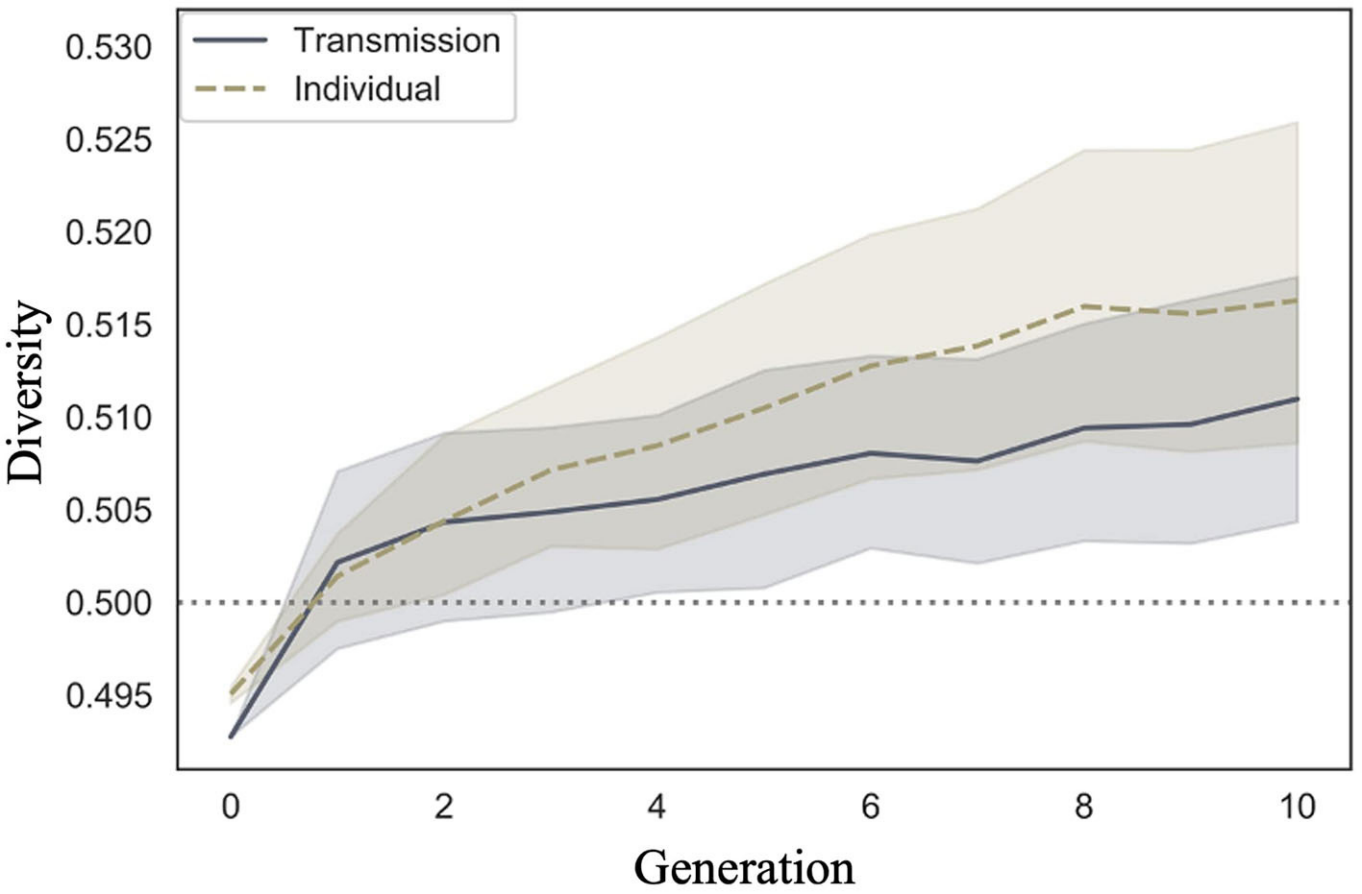
Changes in mean diversity (10 chains in transmission chain; 20 chains in individual condition) over the course of ten generations in Experiment 1.

We examined whether diversity increased across generations in the transmission condition, a replication of Cornish et al. (2013); whether diversity also increased in our individual condition; and whether there were differences in the rate of decrease across conditions. Figure 9 shows the mean diversity values and 95% confidence intervals under each condition and generation. Under both conditions, the diversity increased as the number of generations increased.

The WAIC (−2,309.741) for Model 2 with the interaction included was smaller than the WAIC (−2,304.816) for Model 1 without the interaction, indicating greater predictive power. The difference in the WAIC between the two models was 4.92 (dSE = 5.70), which is not sufficiently large. The Bayesian estimation results for Model 2 (Table 4) showed that the 89% confidence intervals for the generation and interaction terms did not include zero, but the estimates for the interaction term were very close to zero (generation: β = 0.001, CI = [0.001, 0.002]; interaction: β = 0.000, CI = [0.000, 0.001]). In other words, diversity increased with increasing generations under both conditions, but the slopes did not differ between conditions.

**Table 4:** Bayesian estimated values for Model 2 (with interaction) with the depth of diversity in Experiment 1 as dependent variable.

	**β**	***Std***	***Lower 0.89***	***Upper 0.89***	***n_eff***	***Rhat***
Intercept	0.499	0.004	0.493	0.505	1,793.94	1.004
Condition	0.000	0.005	−0.007	0.008	1,707.70	1.004
Generation	0.001	0.000	0.001	0.002	57,558.54	1.000
Interaction	0.000	0.000	0.000	0.001	57,133.02	1.000

**Figure 10:**
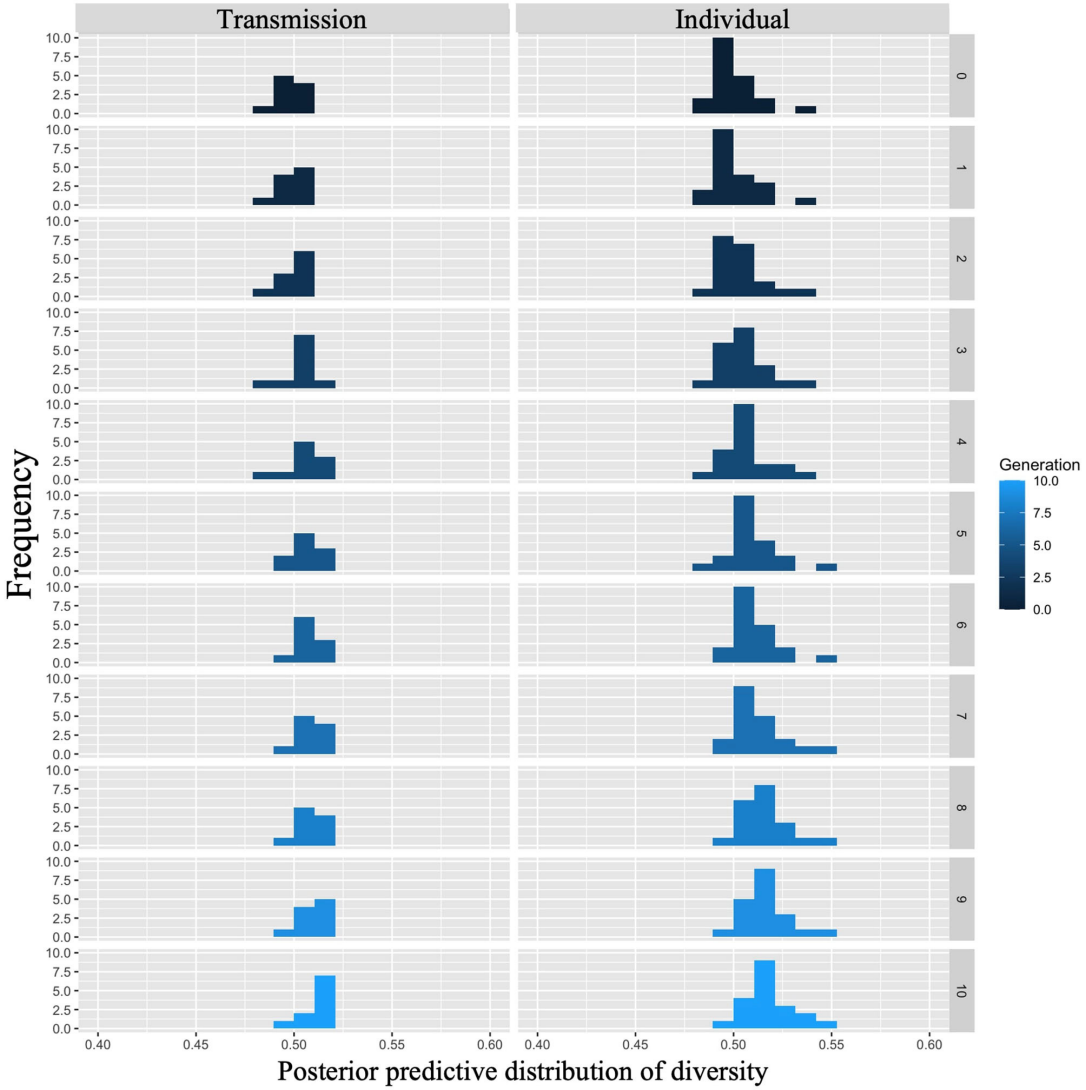
Diversity predicted from posterior distribution of Model 2 in Experiment 1. The **left** column shows the transmission condition, and the **right** column shows the individual condition. Rows indicate generations.

Figure 10 shows the posterior predictive distribution of the interaction model. The rows indicate generations (1–10) and the columns indicate conditions (0: transmission condition, 1: individual condition). The vertical and horizontal axes of each plot indicate the frequency and diversity, respectively. For both conditions, the diversity increased with each generation. In the 10th generation, the distribution of the individual condition was slightly wider to the right, but the mode frequency of the histograms was 0.60 for both conditions.

## 4 Results of Experiment 2

In Experiment 1, the presentation order of the sequences was fixed between chains and generations. This could have contributed to the learnability of the participants in individual conditions. To exclude this possibility and confirm the robustness, we generated new, randomly organized stimulus sequences and conducted Experiment 2, which randomized the presentation order of the sequences among chains and generations. All procedures, except for the presentation order and analysis methods, were identical to those used in Experiment 1.

### 4.1 Accuracy

Supplementary Figure S1 shows the mean accuracy values and 95% confidence intervals under each condition and generation. In both conditions, the accuracy increased as the number of generations increased.

Model 2, which included an interaction term, showed a smaller WAIC (−98,495.45) than Model 1, which did not include an interaction term (WAIC = −98,467.61), indicating a higher predictive power. The difference in WAIC between the two models was 27.84 (dSE = 13.69), which is a sufficiently large difference. The Bayesian estimation results for Model 2 (Supplementary Table S1) showed that 89% confidence intervals for the condition, generation, and interaction terms did not contain zero (condition: β = 0.45, CI = [0.18, 0.73]; generation: β = 0.15, CI = [0.15, 0.16]; interaction: β = 0.03, CI = [0.02, 0.04]). In other words, the accuracy improved with each successive generation in both conditions, but the improvement was greater in the individual condition.

Supplementary Figure S2 shows the distribution of the posterior predictions for Model 2 with interactions. The rows indicate generations (1–10) and the columns indicate conditions (0: transmission condition, 1: individual condition). The vertical and horizontal axes of each plot indicate frequency and accuracy, respectively. Comparing the distribution of the posterior predictions for the first generation, the transmission condition was slightly more accurate than the individual condition; however, the difference was not clear. As the generations progressed, the accuracy of both conditions increased; the individual condition showed higher values. For example, in the 10th generation, the mode of the histogram in the individual condition was 1.00, while it was 0.85 in the transmission condition.

### 4.2 Compression ratio

Supplementary Figure S3 shows the mean compression ratio values and 95% confidence intervals under each condition and generation. Under both conditions, the compression ratio decreased as the number of generations increased.

Model 2, which included an interaction term, showed a smaller WAIC (−26,688.86) compared to Model 1, which did not include an interaction term (WAIC = −26,600.45), indicating a greater predictive power. The difference in WAIC between the two models was 88.41 (dSE = 20.35), which is a sufficiently large difference. The Bayesian estimation results for Model 2 (Supplementary Table S2) showed that the 89% confidence intervals for the generation and interaction terms did not include zero (generation: β = −0.06, CI = [−0.06, −0.05]; interaction: β = 0.02, CI = [0.02, 0.03]). In other words, under both conditions, the compression ratio decreased with increasing generations; however, the degree of decrease was greater under the transmission condition.

The posterior prediction distributions of the interaction model are shown in Supplementary Figure S4. Rows indicate generations (1–10) and columns indicate conditions (0: transmission condition, 1: individual condition). The vertical and horizontal axes of each plot indicate the frequency and compression ratio, respectively. There was little difference in the posterior predictive distribution of the compression ratios in the first generation. As the generation progresses, the compression ratios become smaller in both conditions but are lower in the transmission condition. For example, in the 10th generation, the mode of the histogram in the individual condition was 0.65, while it was 0.60 in the transmission condition.

### 4.3 Depth of hierarchy

Supplementary Figure S5 shows the mean depth of the hierarchy and 95% confidence intervals under each condition and generation. Under both conditions, the depth of the hierarchy increases with the number of generations.

The WAIC (50,751.16) for Model 2 with the interaction included was smaller than the WAIC (50,767.63) for Model 1 without the interaction, indicating greater predictive power. The difference in the WAIC between the two models was 16.47 (dSE = 4.87), which was a small difference. The Bayesian estimation results for Model 2 (Supplementary Table S3) show that the 89% confidence intervals for the generation and interaction terms did not include zero (generation: β = 0.04, CI = [0.03, 0.04]; interaction: β = −0.02, CI = [−0.02, −0.01]). In other words, the hierarchy deepens as generations progress in both conditions, but is deeper in the transmission condition.

The posterior predictive distribution of the interaction model is shown in Supplementary Figure S6. The rows indicate generations (1–10) and the columns indicate conditions (0: transmission condition, 1: individual condition). The vertical axis of each plot indicates the frequency, and the horizontal axis indicates the depth of the hierarchy. The posterior predictive distribution of the hierarchical depth for the first generation was not very different. As the generations progress, the hierarchy deepens under both conditions, but the transmission condition shows a greater increase. For example, in the 10th generation, 1 remained the mode for the depth of the hierarchy in the individual condition, while 2 was the mode in the transmission condition.

### 4.4 Diversity

Supplementary Figure S7 shows the mean diversity values and 95% confidence intervals under each condition and generation. Under both conditions, the diversity increased as the number of generations increased.

The WAIC (−3,427.173) for Model 2 with the interaction included was smaller than the WAIC (−3,422.743) for Model 1 without the interaction, indicating greater predictive power. The difference in the WAIC between the two models was 4.43 (dSE = 5.36), which was not sufficiently large. The Bayesian estimation results for Model 2 (Supplementary Table S4) showed that 89% confidence intervals for the generation and interaction terms did not include zero (generation: β = 0.002, CI = [0.001, 0.002]; interaction: β = 0.001, CI = [0.000, 0.001]). In other words, diversity in both conditions increases with advancing generations, but the individual conditions are more diverse.

The posterior predictive distribution of the model with interactions is shown in Supplementary Figure S8. The rows indicate generations (1–10) and the columns indicate conditions (0: transmission condition, 1: individual condition). The vertical and horizontal axes of each plot indicate the frequency and diversity, respectively. The diversity of both conditions increased as the generations progressed; however, the diversity of the individual conditions increased with each generation. For example, in the 10th generation, the mode for the transmission condition was 0.60, whereas that for the individual condition was 0.65.

## 5 Discussion

In this study, we examined the effects of cultural transmission on the emergence of hierarchical structures through two laboratory experiments using nonverbal tasks. In addition to the transmission conditions, in a replication of Cornish et al. (2013), we used an individual condition without cultural transmission. While Cornish et al. (2013) qualitatively analyzed the emergence of hierarchical structures, we quantitatively analyzed the depth of the hierarchical structures that emerged. We conducted two experiments to verify the robustness of our findings.

**Figure 11:**
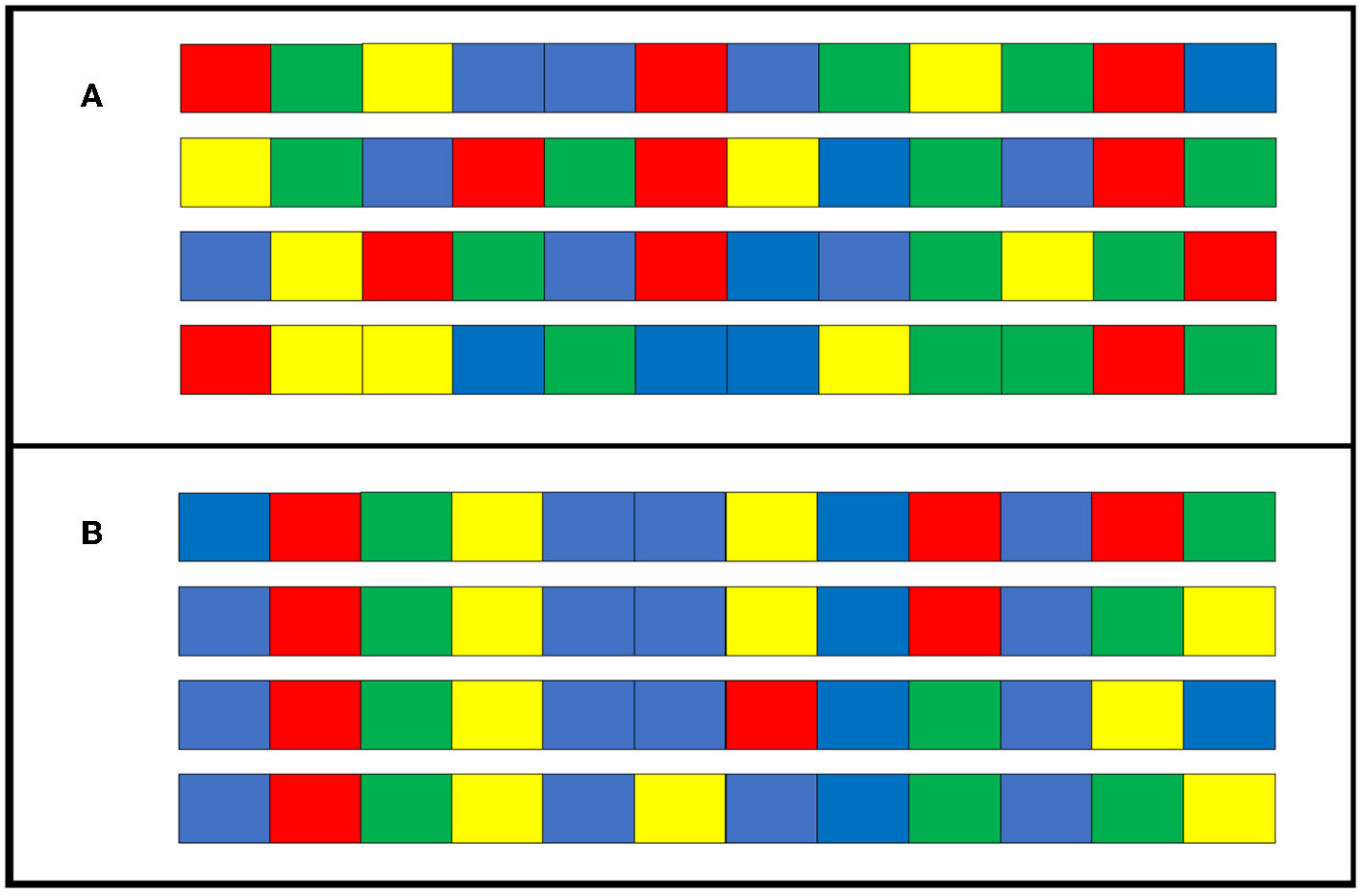
Example sequences from a chain of individual condition in Experiment 1. **(A)** A sample of four initially seeded sequences at generation 0. **(B)** Sequences when the four in **(A)** change slightly between generations and are reproduced at generation 10.

We found that the transmission condition replicated the findings of Cornish et al. (2013), indicating that structures, such as repetition and hierarchical structures, emerge gradually over generations. Compared to the individual condition, in which a single individual repeated learning and reproduction without generational turnover, more structuring occurred in the transmission condition, in which cultural transmission was repeated among several different individuals. However, participants in the individual condition reproduced the stimulus sequence more accurately than those in the transmission condition. Furthermore, the degree of diversification was higher under the individual condition. In other words, the stimulus sequences in the individual condition may have produced unique patterns for each participant that differed from the repetition and hierarchical structures. Figure 11 shows how four of the sequences used in generation 0 (seed) of the individual condition changed after 10 generations. As a result of iterative modification by only one individual, the sequences at generation 10 share common parts. As cognitive biases vary slightly between individuals, unsurprisingly, the easily remembered patterns differ for each individual. These small differences may have been amplified by repeated reconstructions within individuals, resulting in greater diversity under the individual condition than under the transmission condition. On the other hand, there is a common tendency to reduce the randomness of sequences. In general, the sequences with the highest randomness do not have a hierarchical structure. Reducing the randomness of sequences produces hierarchical structure. In addition, sequences with hierarchical structure have multiple chunks, which are generally easier for people to remember (Mathy et al., 2016). Therefore, cultural selection works when sequences are culturally transmitted: hierarchical structures are more likely to be preserved, while other patterns are more likely to be lost. Through repeated cultural selection process, hierarchical structures evolve cumulatively. In Experiment 2, we aimed to replicate the results of Experiment 1 while eliminating the possible influence of presentation order on the stimulus sequences. Experiment 2 replicated the results of Experiment 1 and demonstrated its robustness.

Based on these results, in the transmission condition, the structure likely emerged as a result of the cumulative cultural evolution of stimulus sequences with general features that were easily remembered by many individuals through iterated cultural transmission between different people. Therefore, structural features in the human language, such as hierarchical structures, are likely to evolve cumulatively through iterated cultural transmission among different individuals. This finding supports the claim of computational evolutionary linguistics that learnability promotes the cumulative cultural evolution of structure (Kirby et al., 2008) and extends this traditional claim to cultures other than language. Furthermore, it provides a detailed explanation that the structural features of human culture evolve cumulatively through iterated transmission among different individuals who share and inherit the culture and thus become, on average, more likely to be learned. Furthermore, the finding that cultural transmission can emerge as a structure may not apply to groups of people in which there is little diversity in biases toward learning and transmission. It has been reported that a compositional structure is less likely to emerge in iterated learning involving only children (Raviv and Arnon, 2018). These results could potentially be attributed to the fact that children have not yet developed a bias toward structure, and there is less diversity in biases among individuals. The relationship between the various biases held by people and the emergence of structures is not well understood, and further research is required.

In this study, we have shown that hierarchical structures can arise from even minimal processes of cultural evolution: learning and transmission. This may explain why, in addition to language, similar hierarchical structures are found in different human cultures. In the case of our cultures, even more diverse and complex structures can emerge due to the demands of functions such as expression and convenience. For example, to study the cumulative cultural evolution of the grammars of human languages, it is necessary to introduce into the experiment the requirement of expressing meaning with sequences (Saldana et al., 2019b).

In our experiment, we conducted an individual condition in addition to replicating Cornish et al.'s (2013) transmission condition to manipulate the presence of cultural transmission. However, the individual conditions may have unintended effects. As the total number of trials per participant in the individual condition was much higher than in the transmission condition (300 trials), we interspersed breaks every 50 trials. However, it was not possible to completely eliminate the effects of fatigue or reduce the concentration. Although accuracy increased in the individual condition, the break itself may have had some effect on the memory and replay of the stimulus sequence. To test the hypothesis that cultural transmission among diverse populations promotes the cumulative cultural evolution of hierarchical structures, future modifications to the experimental design will be necessary. For example, one could compare a ten-participant transmission chain in the transmission condition of this study with a half-participant transmission chain (five participants), which would require twice as many stimuli per participant (120 trials) as a ten-participant transmission chain. With 120 trials per person, it was possible to complete the task without breaks. If this hypothesis is correct, the five-person transmission chain experiment may reveal a cumulative cultural evolution of hierarchical structures that are intermediate between the transmission and individual conditions of this study.

Although our study used a grammatical compression algorithm to quantitatively analyze the cumulative cultural evolution of hierarchical structure depth, it examined only a small subset of the structures present in real human culture. Most of the hierarchical structures that emerged in this study had shallow hierarchies with depths of 1–2. One possible explanation for this limitation is the short length of the stimulus sequence, which was set at 12. A longer stimulus sequence was required to facilitate the emergence of deeper hierarchical structures. In addition, longer transmission chains may be necessary for the cumulative development of deeper hierarchical structures in the culture. In this study, the experiment was conducted over 10 generations, but the number of stimulus sequences to be learned was reduced to half the number of participants in the transmission condition because of concerns about the participant load in the individual condition. By manipulating the number of individuals in the transmission chain as described above, experiments can be conducted over a period longer than 10 generations. The “sequiter” algorithm used in our study could also be applied to artificial language learning tasks. Previous iterated learning experiments have focused primarily on the quantitative analysis of compositional structures (Kirby et al., 2008, 2015; Raviv and Arnon, 2018), with only a qualitative analysis of hierarchical structures (Saldana et al., 2019b). Applying a quantitative analysis of the hierarchical structure, which is a prominent feature of human language, to experiments with more realistic language-like tasks would provide further insights into the origins of language structures. For instance, iterated learning experiments can be conducted on non-human animals (Claidière et al., 2014; Saldana et al., 2019a), enabling the direct comparison of emergent structures across species.

## Data availability statement

The datasets presented in this study can be found in online repositories. The names of the repository/repositories and accession number(s) can be found below: https://github.com/SeiyaNAKATA/SimonGameExperiments.

## Ethics statement

The studies involving humans were approved by Center for Experimental Research in Social Sciences, Hokkaido University. The studies were conducted in accordance with the local legislation and institutional requirements. The participants provided their written informed consent to participate in this study.

## Author contributions

MT and SN designed the experiments, analyzed the data, and wrote the manuscript. SN constructed the experimental program and conducted the experiments. All authors contributed to the article and approved the submitted version.
