# Supplementary material for: Hierarchical structures emerge from the cultural transmission: an iterated learning experiment using a non-linguistic task
Source: Front Artif Intell. 2023 Dec 22;6:1221329. doi: 10.3389/frai.2023.1221329 (PMC10770839; doi:10.3389/frai.2023.1221329)
Supplement: Supplementary file 1 [file Data_Sheet_1.docx]

Supplementary Material

# Model formulae

We constructed Bayesian multilevel models with condition, generation and their interaction as independent variables. All the models were analyzed with rethinking package ver. 2.31 (<https://github.com/rmcelreath/rethinking>). Here we include each Model 2 formula (with the interaction between condition and generation) used for each analysis in the main article.

## Accuracy

m.acc.Beta.exp1 <- ulam(

alist(

Accuracy_mod ~ dbeta(at, bt),

at <- pbar * theta,

bt <- (1 - pbar) * theta,

pbar <- inv_logit(a + a_chain[Chain] + a_sequence[Sequence] + b_C * Condition + b_G * Generation + b_CG * Condition * Generation),

a ~ dnorm(0, 1),

c(b_C, b_G, b_CG) ~ dnorm(0, 1),

a_chain[Chain] ~ dnorm(0, sigma_chain),

a_sequence[Sequence] ~dnorm(0, sigma_sequence),

sigma_chain ~ dcauchy(0, 1),

sigma_sequence ~ dcauchy(0, 1),

theta ~ dexp(1)

),

data=df.noGen0[df.noGen0$Experiment==1,], warmup=1000, iter=10000, chains=4, cores=4, log_lik=TRUE

)

## Compression ratio

m.comp.Beta.exp1 <- ulam(

alist(

Compression_ratio_mod ~ dbeta(at, bt),

at <- pbar * theta,

bt <- (1 - pbar) * theta,

pbar <- inv_logit(a + a_chain[Chain] + a_sequence[Sequence] + b_C * Condition + b_G * Generation + b_CG * Condition * Generation),

a ~ dnorm(0, 1),

c(b_C, b_G, b_CG) ~ dnorm(0, 1),

a_chain[Chain] ~ dnorm(0, sigma_chain),

a_sequence[Sequence] ~dnorm(0, sigma_sequence),

sigma_chain ~ dcauchy(0, 1),

sigma_sequence ~ dcauchy(0, 1),

theta ~ dexp(1)

),

data=df[df$Experiment==1,], warmup=1000, iter=10000, chains=4, cores=4, log_lik=TRUE

)

## Depth of hierarchy

m.dep.Pois.exp1 <- ulam(

alist(

Depth_of_hierarchy1 ~ dpois(lambda),

log(lambda) <- a + a_chain[Chain] + a_sequence[Sequence] + b_C * Condition + b_G * Generation + b_CG * Condition * Generation,

a ~ dnorm(0, 1),

c(b_C, b_G, b_CG) ~ dnorm(0, 1),

a_chain[Chain] ~ dnorm(0, sigma_chain),

a_sequence[Sequence] ~dnorm(0, sigma_sequence),

sigma_chain ~ dcauchy(0, 1),

sigma_sequence ~ dcauchy(0, 1)

),

data=df[df$Experiment==1,], warmup=1000, iter=10000, chains=4, cores=4, log_lik=TRUE

)

## Diversity

m.iden.Gauss.exp1 <- map2stan(

alist(

Identifiability ~ dnorm(mu, sigma),

mu <- a + a_chain[Chain] + b_C * Condition + b_G * Generation + b_CG * Condition * Generation,

c(a, b_C, b_G, b_CG) ~ dnorm(0, 10),

a_chain[Chain] ~ dnorm(0, sigma_chain),

sigma ~ dunif(0,50),

sigma_chain ~ dcauchy(0, 1)

),

data=df[df$Experiment==1,], warmup=1000, iter=10000, chains=4, cores=4

)

# Supplementary Figures and Tables for Experiment 2

We conducted two experiments to confirm the robustness of our findings. In Experiment 1, the order of presentation of the stimulus sequences was fixed across chains and across generations. In Experiment 2, the order in which the stimulus sequences were presented was randomized and was modified to differ between generations and transmission chains. Here we include the figures and tables which shows the results in Experiment 2. Figures show the data and posterior predictive distribution of each measure in Experiment 2. Tables shows Bayesian estimated values with each measure in Experiment 2.

## Accuracy for Experiment 2


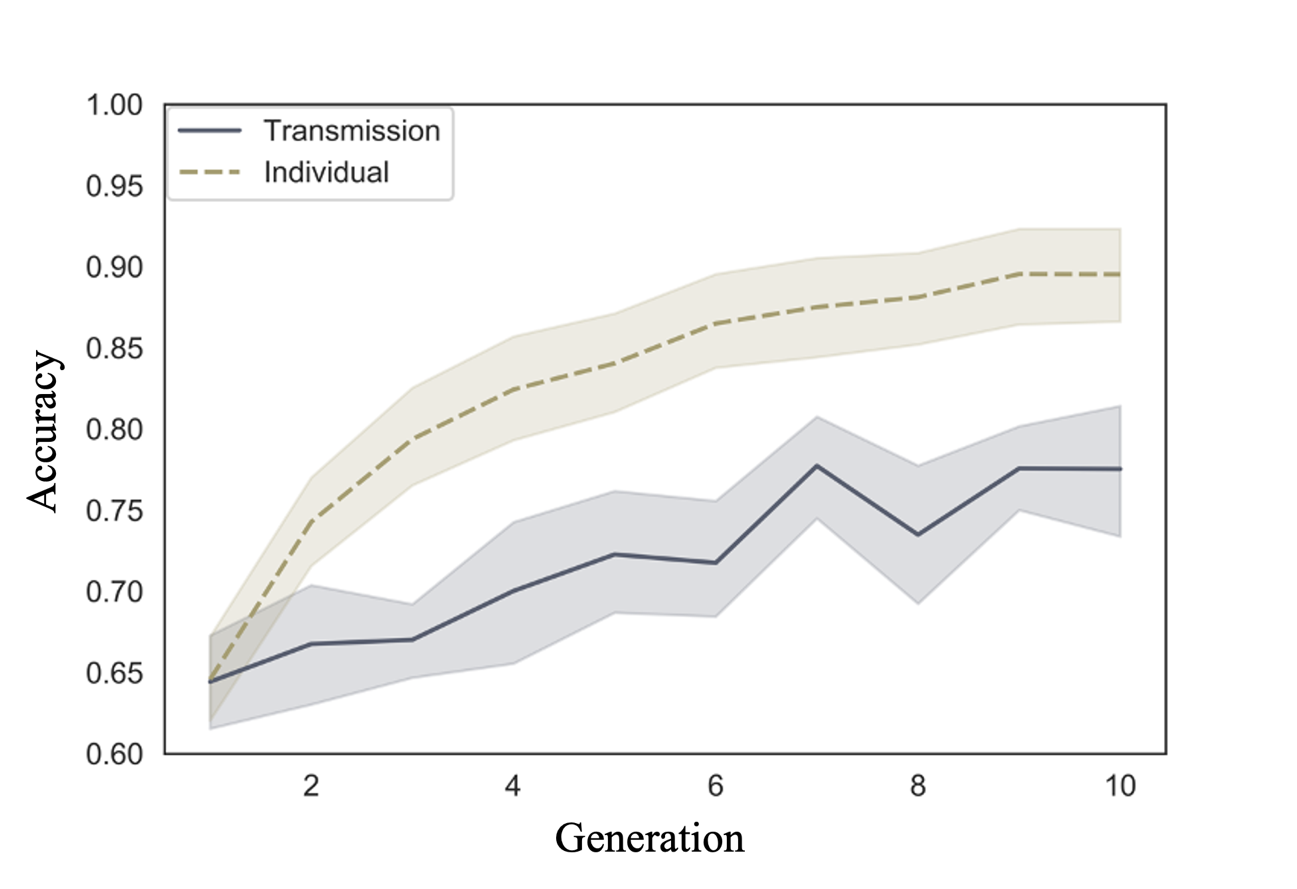


Figure S1 Changes in mean accuracy (10 chains in transmission chain; 20 chains in individual condition) over the course of ten generations in Experiment 2.

Table S1 Bayesian estimated values for Model 2 (with interaction) with accuracy in Experiment 2 as dependent variable.

|  | *β* | *Std* | *Lower 0.89* | *Upper 0.89* | *n_eff* | *Rhat* |
| --- | --- | --- | --- | --- | --- | --- |
| Intercept | 0.52 | 0.14 | 0.30 | 0.75 | 4043.38 | 1.00 |
| Condition | 0.45 | 0.17 | 0.18 | 0.73 | 3413.67 | 1.00 |
| Generation | 0.15 | 0.00 | 0.15 | 0.16 | 50215.21 | 1.00 |
| Interaction | 0.03 | 0.01 | 0.02 | 0.04 | 52582.03 | 1.00 |


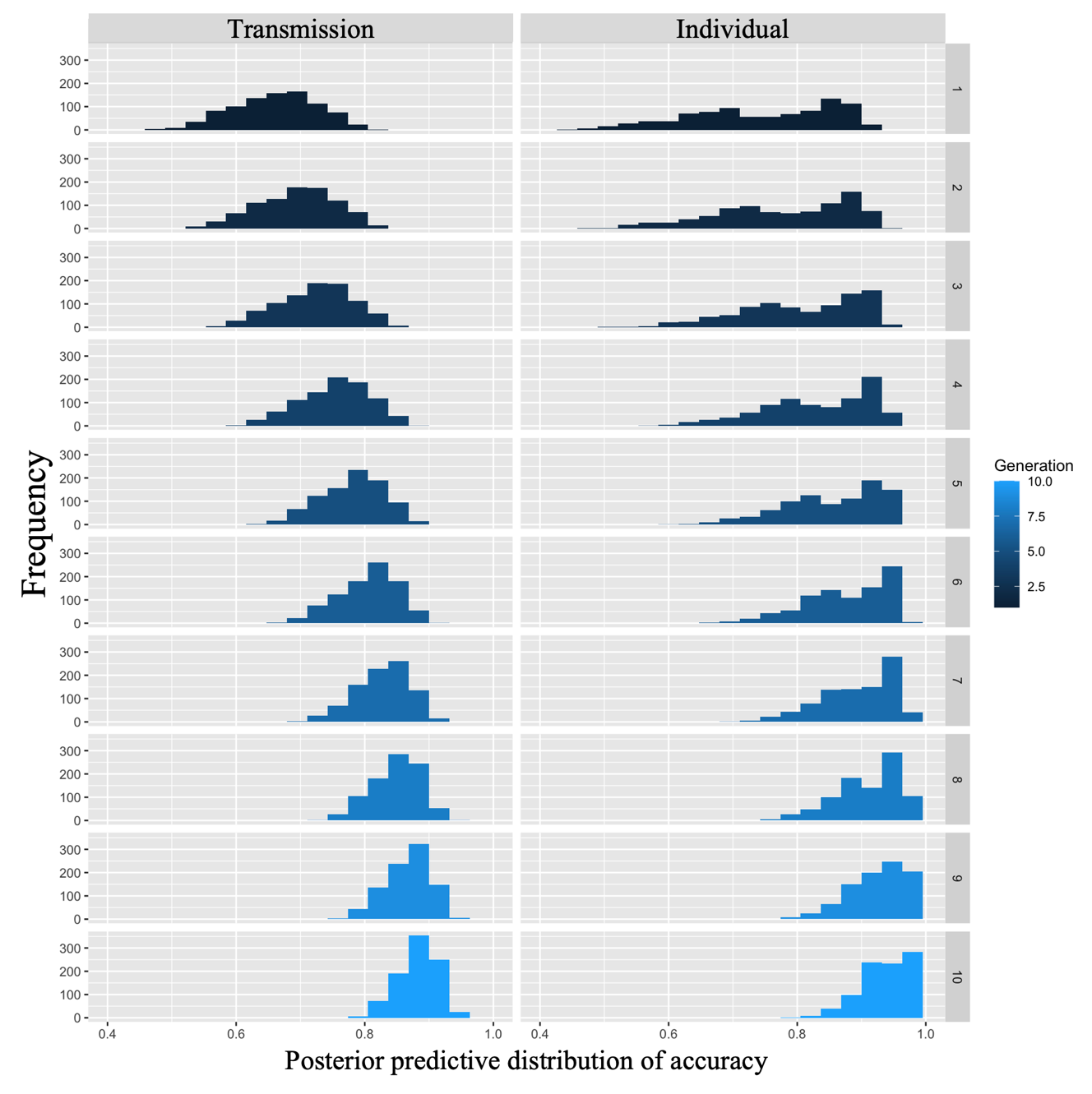


Figure S2 Accuracy predicted from posterior distribution of Model 2 in Experiment 2. The left column shows the transmission condition, and the right column shows the individual condition. Rows indicate generations.

## Compression ratio for Experiment 2


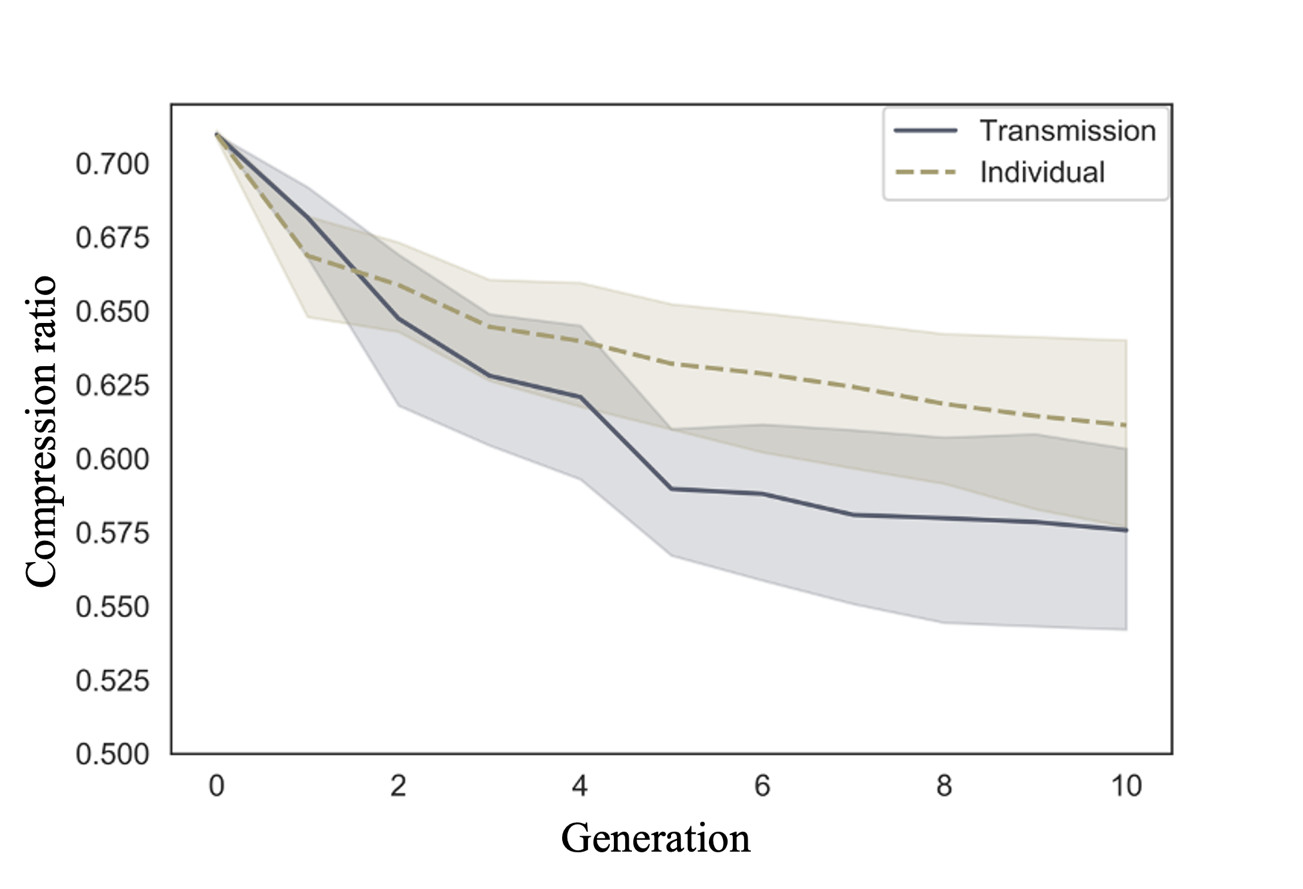


Figure S3 Changes in mean compression ratio (10 chains in transmission chain; 20 chains in individual condition) over the course of ten generations in Experiment 2.

Table S2 Bayesian estimated values for Model 2 (with interaction) with compression ratio in Experiment 2 as dependent variable.

|  | *β* | *Std* | *Lower 0.89* | *Upper 0.89* | *n_eff* | *Rhat* |
| --- | --- | --- | --- | --- | --- | --- |
| Intercept | 0.73 | 0.06 | 0.64 | 0.82 | 1801.04 | 1.00 |
| Condition | 0.00 | 0.07 | –0.11 | 0.12 | 1781.64 | 1.00 |
| Generation | –0.06 | 0.00 | –0.06 | –0.05 | 53229.12 | 1.00 |
| Interaction | 0.02 | 0.00 | 0.02 | 0.03 | 58485.38 | 1.00 |


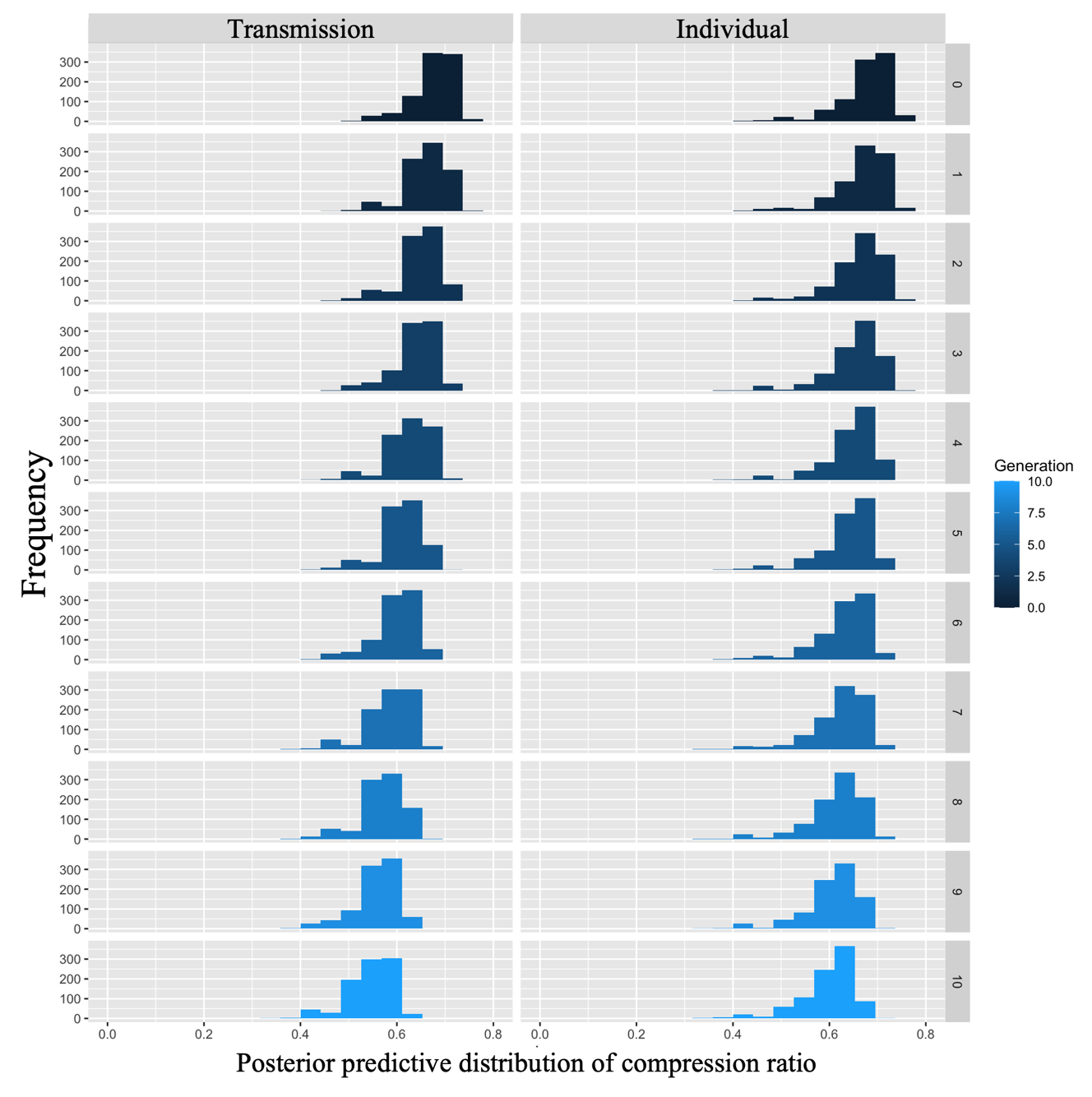


Figure S4 Compression ratio predicted from posterior distribution of Model 2 in Experiment 2. The left column shows the transmission condition, and the right column shows the individual condition. Rows indicate generations.

## Depth of hierarchy for Experiment 2


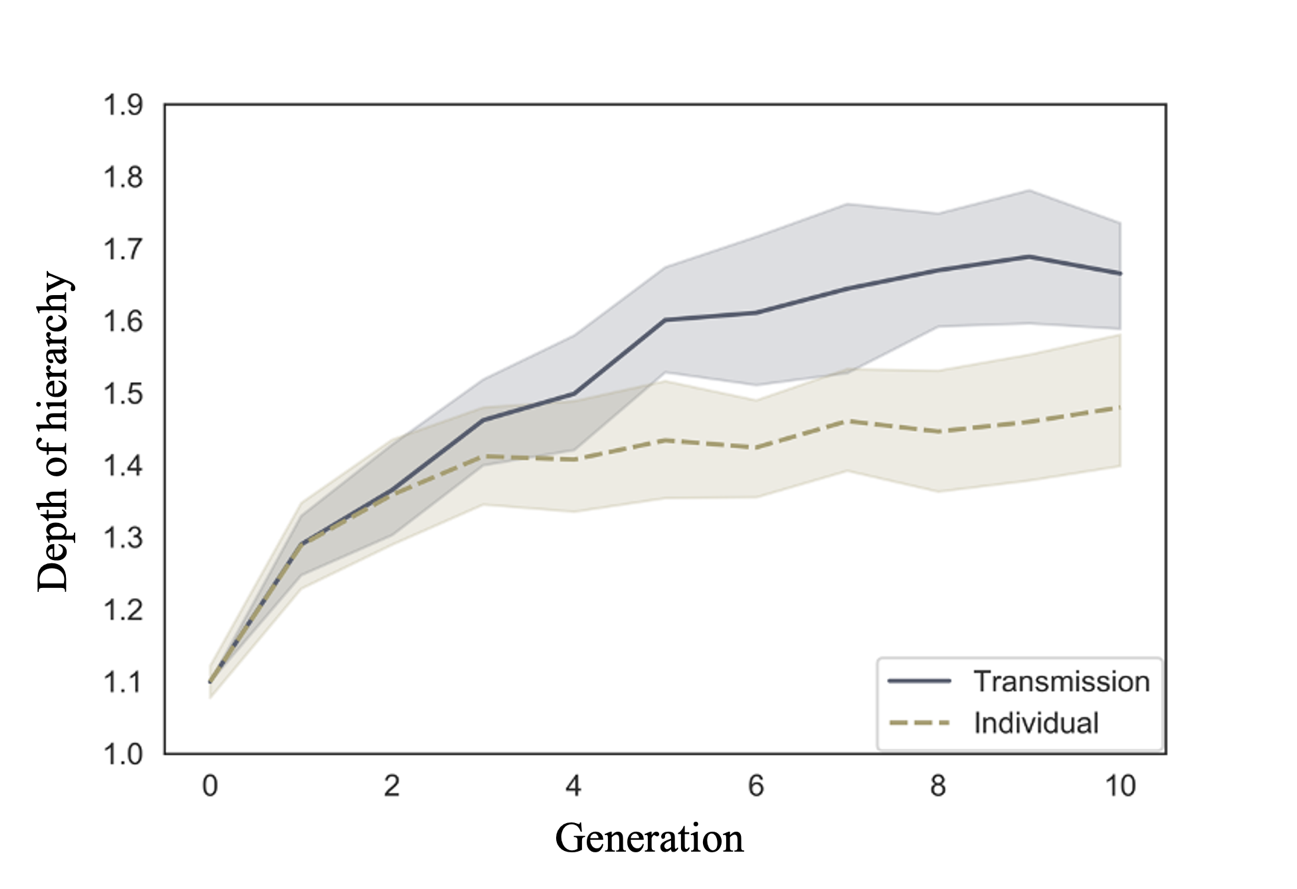


Figure S5 Changes in mean depth of hierarchy (10 chains in transmission chain; 20 chains in individual condition) over the course of ten generations in Experiment 2.

Table S3 Bayesian estimated values for Model 2 (with interaction) with the depth of hierarchy in Experiment 2 as dependent variable.

|  | *β* | *Std* | *Lower 0.89* | *Upper 0.89* | *n_eff* | *Rhat* |
| --- | --- | --- | --- | --- | --- | --- |
| Intercept | 0.22 | 0.03 | 0.18 | 0.27 | 6334.08 | 1.00 |
| Condition | –0.00 | 0.04 | –0.06 | 0.06 | 7276.94 | 1.00 |
| Generation | 0.04 | 0.00 | 0.03 | 0.04 | 38088.83 | 1.00 |
| Interaction | –0.02 | 0.00 | –0.02 | –0.01 | 36126.58 | 1.00 |


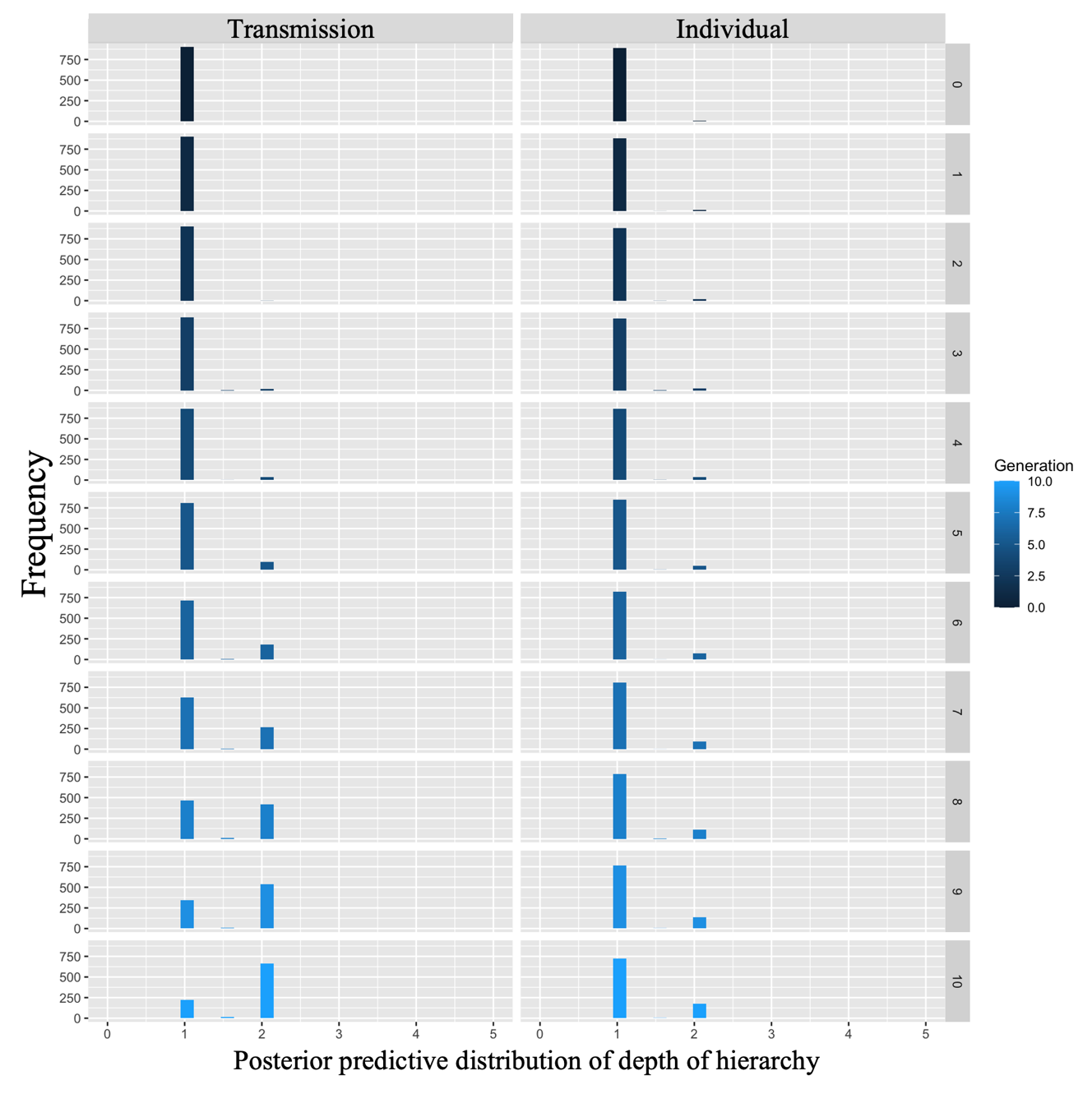


Figure S6 Depth of hierarchy predicted from posterior distribution of Model 2 in Experiment 2. The left column shows the transmission condition, and the right column shows the individual condition. Rows indicate generations.

## Diversity for Experiment 2


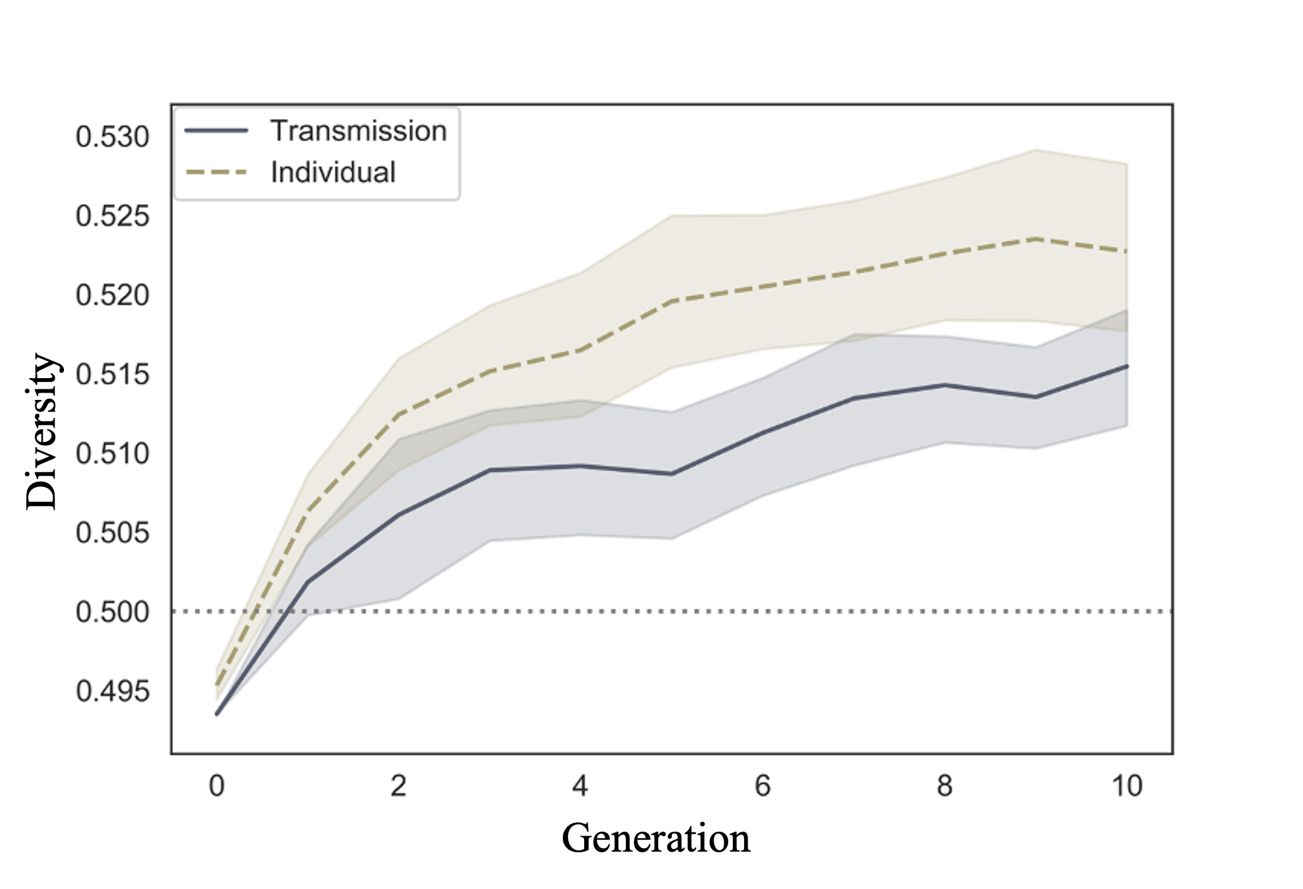


Figure S7 Changes in mean diversity (10 chains in transmission chain; 20 chains in individual condition) over the course of ten generations in Experiment 2.

Table S4 Bayesian estimated values for Model 2 (with interaction) with the depth of diversity in Experiment 2 as dependent variable.

|  | *β* | *Std* | *Lower 0.89* | *Upper 0.89* | *n_eff* | *Rhat* |
| --- | --- | --- | --- | --- | --- | --- |
| Intercept | 0.500 | 0.003 | 0.496 | 0.504 | 4697.56 | 1.000 |
| Condition | 0.005 | 0.003 | –0.000 | 0.010 | 4356.49 | 1.000 |
| Generation | 0.002 | 0.000 | 0.001 | 0.002 | 47153.75 | 1.000 |
| Interaction | 0.001 | 0.000 | 0.000 | 0.001 | 50139.56 | 1.000 |


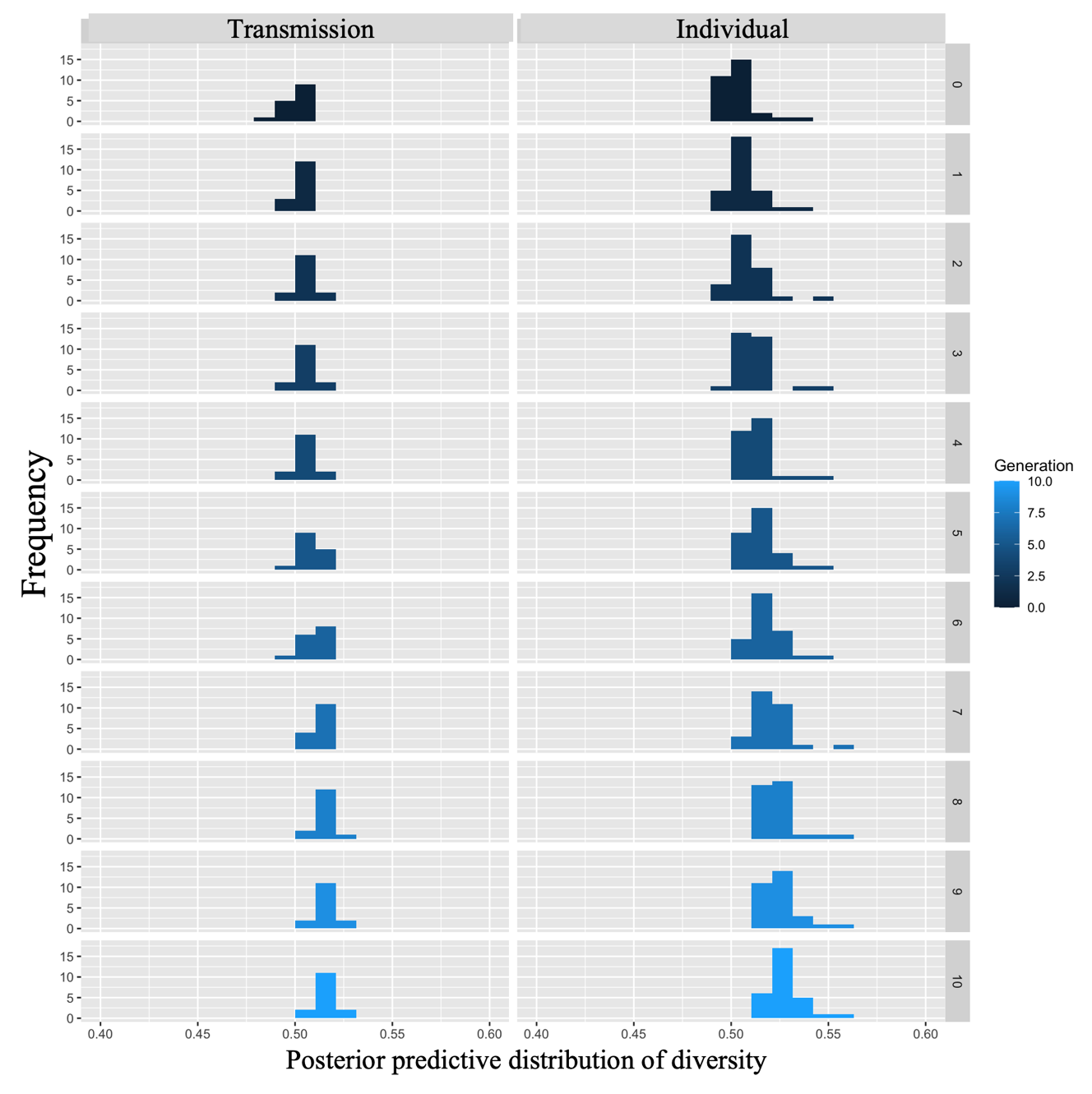


Figure S8 Diversity predicted from posterior distribution of Model 2 in Experiment 2. The left column shows the transmission condition, and the right column shows the individual condition. Rows indicate generations.
